# Inertial Microfluidics Enabling Clinical Research

**DOI:** 10.3390/mi12030257

**Published:** 2021-03-03

**Authors:** Srivathsan Kalyan, Corinna Torabi, Harrison Khoo, Hyun Woo Sung, Sung-Eun Choi, Wenzhao Wang, Benjamin Treutler, Dohyun Kim, Soojung Claire Hur

**Affiliations:** 1 Department of Mechanical Engineering, Johns Hopkins University, 3400 N Charles Street, Baltimore, MD 21218, USA; skalyan6@jhu.edu (S.K.); ctorabi1@jhu.edu (C.T.); harrison.khoo@jhu.edu (H.K.); s.choi@jhu.edu (S.-E.C.); 2 Department of Chemical and Biomolecular Engineering, Johns Hopkins University, 3400 N Charles Street, Baltimore, MD 21218, USA; hsung10@jhu.edu; 3 Department of Biomedical Engineering, Johns Hopkins University, 3400 N Charles Street, Baltimore, MD 21218, USA; wwang120@jhu.edu (W.W.); btreutl1@jhu.edu (B.T.); 4 Department of Mechanical Engineering, Myongji University, Yongin-si 17508, Korea; 5 Institute for NanoBioTechnology, Johns Hopkins University, 3400 N Charles Street, Baltimore, MD 21218, USA; 6 Department of Oncology, Johns Hopkins University, 600 N Wolfe St, Baltimore, MD 21205, USA; 7 Sidney Kimmel Comprehensive Cancer Center, Johns Hopkins University, 401 N Broadway, Baltimore, MD 21231, USA

**Keywords:** inertial microfluidics, cell purification, translational research, clinical research, sample processing, hybrid devices, high throughput

## Abstract

Fast and accurate interrogation of complex samples containing diseased cells or pathogens is important to make informed decisions on clinical and public health issues. Inertial microfluidics has been increasingly employed for such investigations to isolate target bioparticles from liquid samples with size and/or deformability-based manipulation. This phenomenon is especially useful for the clinic, owing to its rapid, label-free nature of target enrichment that enables further downstream assays. Inertial microfluidics leverages the principle of inertial focusing, which relies on the balance of inertial and viscous forces on particles to align them into size-dependent laminar streamlines. Several distinct microfluidic channel geometries (e.g., straight, curved, spiral, contraction-expansion array) have been optimized to achieve inertial focusing for a variety of purposes, including particle purification and enrichment, solution exchange, and particle alignment for on-chip assays. In this review, we will discuss how inertial microfluidics technology has contributed to improving accuracy of various assays to provide clinically relevant information. This comprehensive review expands upon studies examining both endogenous and exogenous targets from real-world samples, highlights notable hybrid devices with dual functions, and comments on the evolving outlook of the field.

## 1. Introduction

Translational research advances scientific discoveries to practical settings, offering tangible patient and public health benefits. To accomplish this, innovative technologies must accurately analyze complex real-world samples in relevant time periods. High-throughput technologies enable the collection and analysis of a large amount of health data to make timely clinical decisions based on reproducible and statistically significant results. When coupled with easily obtainable samples, such as blood or urine, these technologies permit routine sampling in a timely manner to track a patient’s overall health conditions. These advantages are particularly important to the processing of biological samples with high background signals, where the viability and integrity of the processed sample are essential for performing subsequent analysis.

**Figure 1:**
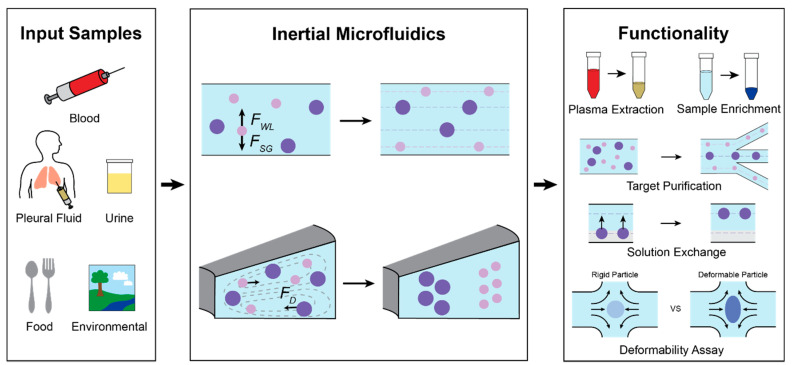
Depiction of concept and analytical capabilities of inertial microfluidics.

Inertial microfluidics (IM) is an effective translational technology that has proved capable of analyzing real, non-idealized biological and environmental fluid samples (Figure 1). Inertial focusing, the phenomenon that IM utilizes, relies on the balance of hydrodynamic forces to order particles into distinct streamlines within a microchannel based on flowing particles’ intrinsic physical properties. The physics of IM operation and manipulation of relevant forces have been extensively reviewed and can be found elsewhere [1,2,3,4]. In brief, IM utilizes hydrodynamic lift forces arising within microchannels operating at finite Reynolds number (Re > 1), or the ratio of inertial to viscous forces. Competing wall effect lift, shear gradient lift, and Dean drag forces equilibrate flowing particles at different streamlines depending on the properties of individual particles within a complex solution. IM devices are tailored to control these forces and isolate, trap, or concentrate particles with specific physical characteristics. Often, high flow rates are used to ensure the generation of relevant hydrodynamic forces, which provides the added benefit of faster sample processing.

**Figure 2:**
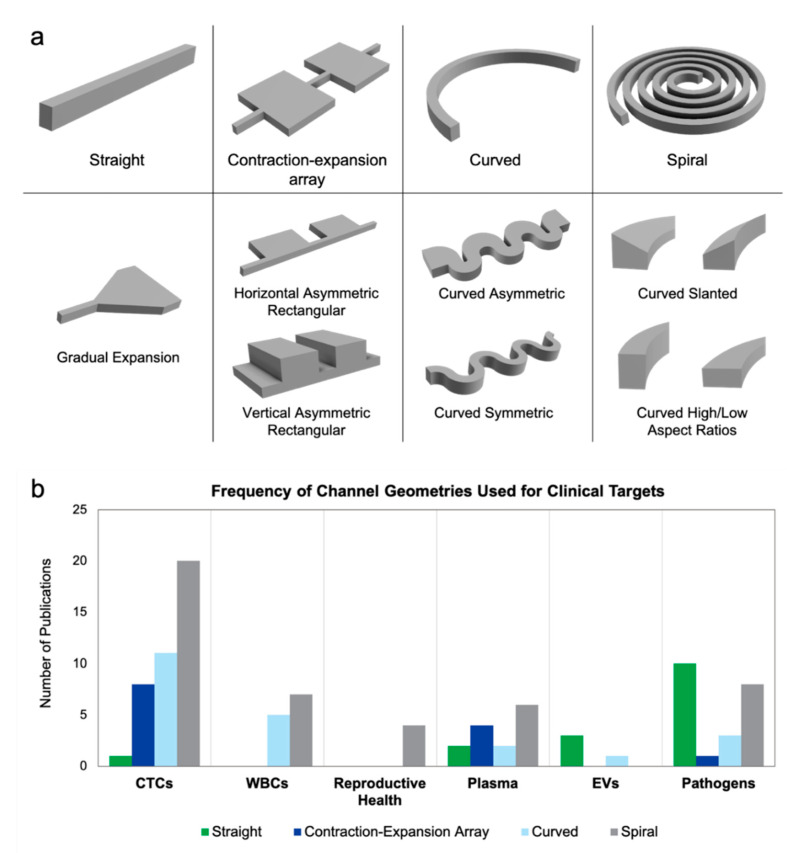
(**a**) Channel geometries frequently used in inertial microfluidics. Several variations of straight, contraction-expansion array, curved, and spiral geometries are used to manipulate particles into different streamlines. (**b**) Summary of channel geometries used for the analysis of clinical targets investigated in this review. Targets investigated are white blood cells (WBCs), circulating tumor cells (CTCs), reproductive health-related particles, extracellular vesicles (EVs), blood plasma, and pathogens.

Channel geometry and operating conditions can be fine-tuned to achieve application-specific particle manipulation. Figure 2a illustrates four general categories of IM geometries that have been implemented: straight, contraction-expansion array, curved, and spiral channels. Straight channels rely on the interplay of two primary lateral forces, the wall effect lift force, *F*_WL_, and shear gradient lift force, *F*_SG_ (Figure 1) [5,6]. The relationship of these forces is summarized as $F_{L}=\frac{\rho U^{2}a^{4}}{H^{2}}$, where ρ is the fluid density, U is the maximum velocity, a is the particle diameter, and H is the channel width [2,6]. These two inertial lift forces cause particles to migrate into distinct equilibrium focusing positions, primarily based on the particle diameter. Size-dependent equilibrium positions can be enhanced with a viscoelastic carrier fluid to improve separation resolution sufficiently to manipulate submicron particles [7]. A region of gradual expansion placed downstream of inertial focusing can create greater separation among particle focusing streamlines, allowing for higher purity separation of particle populations.

Other geometries generate secondary flows that create additional hydrodynamic effects beyond F_WL_ and F_SG_ for improved particle manipulation. Dean flow is a secondary flow that produces counter-rotating vortices that form perpendicular to the bulk flow direction. This creates a Dean drag force (F_D_) on particles in the flow, causing lateral migration, dependent on their size and the flow velocity (Figure 1). Contraction-expansion arrays (CEAs), whose cross-sections periodically widen and narrow, utilize Dean drag forces to differentiate the focusing positions of particles depending on their sizes [8]. Furthermore, the recirculating flow created in the expanding chamber of CEA at high flow rates (Re >100) has been employed to selectively trap particles above a set size threshold, enabling size-based hydrodynamic filtration without physical filter structures [9]. Curved channels also use the Dean drag force to inertially focus particles and they are generally used for applications requiring shorter channel length than straight channels [10,11]. Having the same focusing principle as the curved channels, spiral microchannels provide inertial focusing but in a much smaller footprint [12]. Dean Flow Fractionation (DFF) utilizes the Dean drag force to focus particles of different sizes into distinct streamlines, separating polydisperse particles with high purity in a spiral microchannel [13]. The cross-section of a spiral microchannel can be tuned to further improve the size-resolution of DFF. Each of these geometries provides different advantages that are more critical for certain targets and applications, leading different applications to favor certain geometries (Figure 2b). Unless otherwise noted, IM devices covered in this review have been fabricated using conventional microfabrication and soft lithography or mold-based thermoplastic techniques.

Recent advancements of IM have demonstrated its ability to process complex samples for downstream assays in high throughput while maintaining the viability and integrity of the target particle. These devices enable sensitive assays of rare targets by purifying biological objects from heterogeneous samples and minimizing background noise. Since IM devices can regulate the position of targets within the microchannel using only hydrodynamic forces, IM enables rapid, automated solution exchange without damaging samples and allows for high-speed, precise measurements of individual cell characteristics (e.g., size, deformability). IM technologies focus on targets of a wide size range, including large human cells (~10 µm), pathogenic bacteria, fungus, and parasites (~1 µm), submicron extracellular vesicles (0.1–1 µm), and viruses (~0.1 µm). By providing these functionalities, IM enables fast and accurate diagnosis and prognosis of various diseases, guidance for therapy selection, and assessment of public health risks.

In this review, we highlight technologies that demonstrate clinical utility for sample processing and analysis and are validated using complex samples from patients and environments. First, we discuss technologies that have been used to analyze targets that are endogenous to the human body and carry valuable clinical information. Circulating tumor cells (CTCs), white blood cells (WBCs), reproductive health-related targets, blood plasma, and extracellular vesicles (EVs) provide unique information on patient health and disease pathology. Next, we discuss technologies that probe exogenous targets, namely bacteria, fungi, viruses, and parasites, all of which are pathogenic. Exogenous targets are enriched from blood samples for individual patient data or from environmental samples (e.g., air, water) for public health information and disease prevalence. Finally, we highlight recent notable developments of IM devices for clinical applications, emphasizing hybrid devices, and provide an outlook on the evolving field as it becomes more important to analyzing patient and public health.

## 2. Endogenous Targets

### 2.1. Circulating Tumor Cells

Introduction

Cancer remains a prevalent disease, with projections of 1.9 million new cases and 600,000 deaths in the US in 2021 [14]. Targeted therapeutic treatment options are often complicated by high patient heterogeneity and constant disease mutations that alter target marker expression. While effective for point characterization of cancer, tumor tissue biopsies are too invasive for routine sampling and are insufficient for monitoring metastatic regions [15,16]. Circulating tumor cells (CTCs), found in cancer patients’ blood, are emerging as alternative targets to advise tumor treatment [17,18,19]. Unlike tissue biopsies, CTCs are extracted from blood samples, paving the way for noninvasive, routine sampling. These liquid biopsies can serve as a prognostic guide to help inform clinicians of disease severity. The isolation of rare CTCs from billions of red blood cells (RBCs) and white blood cells (WBCs) is a technical challenge [20]. CellSearch (Veridex), the only existing FDA-approved technology for CTC enumeration, has limited clinical utility because it relies on affinity-based isolation techniques that prevent viable cell capture for further analysis [21].

Inertial microfluidics (IM) is a tested translational technology that exploits cellular size and deformability to isolate CTCs (4–30 µm) from consistently smaller, undesired RBCs (8 µm) and WBCs (7–12 µm) [17,22,23,24]. This separation method is generalizable to different cancer types and produces viable CTCs that preserve their genetic makeup for subsequent analysis. Other label-free CTC isolation technologies, such as filtration, electrokinetic, and acoustophoresis based approaches, have been previously outlined [25,26,27]. Nevertheless, IM remains advantageous for its high sample purity, throughput, simple working principle, and ease of use. Several IM device geometries have been used for CTC isolation from patient samples. The Vortex chip (Vortex Biosciences) [28] and the ClearCell FX system (Clearbridge BioMedics) [13] are two successfully commercialized platforms for CTC isolation that rely on contraction-expansion arrays and spiral microchannels, respectively. Other research platforms include the CTC-iChip, a sorting device combining deterministic lateral displacement (DLD) pillars with asymmetrically curved channel focusing and magnetophoresis [29], and the Labyrinth chip, a microchannel with sharp corners [30]. This section will focus on the downstream assays performed on patient samples and enabled by translational IM devices that expand the significant clinical value of CTCs in studying various types of cancer.

Developing Predictors of Patient Outcome

**Figure 3:**
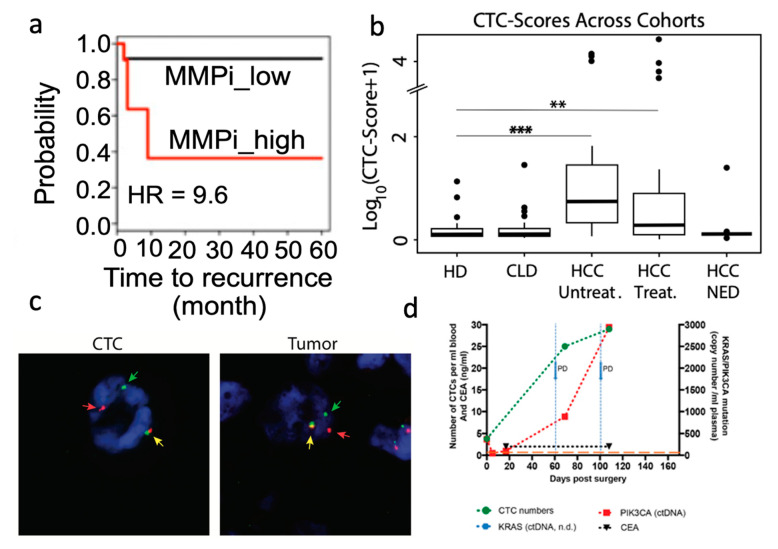
IM CTC purification enables analyses directly beneficial for improving cancer patient health. (**a**) Kaplan–Meier survival curves associated with the MMPi model derived from CTC transcriptomics depict patient likelihood for recurrence-free survival. Reprinted from [31]; permission conveyed through CC BY-NC-ND 4.0. (**b**) Box plot depicting patients with the active disease having higher CTC scores compared to healthy donors (HD), those with chronic lung disease (CLD), and those with no evidence of disease (NED). Reprinted with permission from [34]. (**c**) Break-apart FISH probes revealed identical ALK rearrangements in an NSCLC patient’s CTCs and surgical tumor biopsy. Reprinted from [36]; permission conveyed through CC BY 3.0. (**d**) A longitudinal study of the patient following treatment revealed elevated CTC count and ctDNA mutation copies following evidence of progressive disease (PD). Reprinted from [37]; permission conveyed through CC BY 3.0.

IM CTC isolation provides minimally invasive cancer cell samples for downstream sequencing, enabling longitudinal follow-up studies to monitor disease progression. Data from CTC sequencing depicts mutational profiles that can be aggregated into different scores predictive of patient recovery. Lim et al. passed ClearCell FX-enriched CTCs through a second microfluidic device that separated cells for single-cell sequencing [31]. Analyzing single-cell CTC sequences from non-small-cell lung cancer (NSCLC) patients revealed an improved prognosis of recurrence-free survival (RFS) based on the identification of matrix metalloproteases (MMP) mutations and summarized with the MMP index (MMPi) (Figure 3a). The model led to a 30% reduction in scoring variability and improved stratification of RFS outcomes compared to the existing tumor matrisome index. Miyamoto et al. employed droplet digital polymerase chain reaction (ddPCR) on CTC-iChip isolated CTCs to predict patient outcomes using Androgen Receptor (AR) signal outputs [32]. Expression of AR-related genes led to the subcategorization of “high-risk” metastatic castration-resistant prostate cancer (mCRPC) patients with poor overall survival rate using a scoring system. Similarly, Rzhevskiy et al. isolated localized prostate cancer tumor cells shed into patients’ urine using a slanted spiral microchannel [33]. Subsequent analysis revealed a positive correlation between cancer cell count and both the Gleason score and blood prostate-specific antigen (PSA) levels, the two gold-standard assays for prostate cancer prognosis. Kalinich et al. used a CTC-iChip to isolate CTCs and performed whole transcriptome amplification prior to ddPCR [34]. Transcriptome amplification signals were aggregated into a single CTC score that could accurately classify individuals with active disease (Figure 3b). In addition, combining CTC score analysis with the monitoring of alpha fetoprotein (AFP) levels in serum yielded as high as 86% positive predictive value of early hepatocellular carcinoma (HCC) detection in high-risk patients, which is drastically higher than the 6% positive predictive value of AFP levels alone. A similar CTC score was developed for melanoma using 19 related genes, as determined through CTC-iChip and ddPCR processing of patient samples [35]. Patients with a decrease in score after immune checkpoint inhibition therapies also had better progression-free survival, suggesting a predictor for a disease with few universal markers. These studies illustrate how IM supports downstream sequencing, which has the clinical utility to assess patient conditions.

Guiding Therapeutic Selection and Monitoring Patient Response

IM-isolated CTCs have been screened for proteins directly targetable with therapeutics. In particular, the administration of immunotherapeutic drugs to individuals with tumor expression of programmed death ligand 1 (PD-L1) has been linked to improved patient outcomes over standard chemotherapy [38]. Several groups have used the ClearCell FX platform and Vortex chip to isolate CTCs and stained cells for PD-L1 expression [39,40,41,42]. Dhar et al. found an association between levels of PD-L1 expression in tumor samples and CTCs. 3 out of 4 patients with >50% PD-L1+ CTCs responded well to anti-PD-L1 treatment [40].

Tyrosine Kinase Inhibitors (TKIs) have been demonstrated as effective therapeutics for tumors with epidermal growth factor receptor (EGFR) anomalies, including mutations and amplification [43]. Following enrichment of CTCs in a spiral microchannel, Kulasinghe et al. confirmed EGFR mutational status in 3 out of 3 lung cancer patients with an antibody targeting the exon 19 deletion [41]. Other groups have used fluorescence in situ hybridization (FISH) analysis to identify EGFR gene amplification from CTCs enriched using spiral or labyrinth chips [42,44,45,46]. Yeo et al. used a single-cell capture device to isolate ClearCell FX-enriched CTCs for sequencing [47]. Tumor and liquid biopsies were taken from late-stage NSCLC patients with EGFR TKI resistance. Sequencing revealed identical mutations in both samples, demonstrating the clinical potential of CTCs for monitoring TKI drug response. Onidani et al. compared the genomic profiles of a colorectal cancer patient’s CTCs and circulating tumor DNA (ctDNA) [48]. They showed the utility of both CTC and ctDNA analysis in tracking a patient’s real-time mutational evolution to anti-EGFR therapy, signaling drug resistance.

TKIs have been effective in targeting and treating cancers with specific anaplastic lymphoma kinase (ALK) rearrangements [49]. Several studies have incorporated cytogenetic FISH assays with break-apart probes to visualize ALK rearrangements in the lung, breast, and head and neck cancer CTCs [36,41,42,44,50]. Identical rearrangement patterns were visualized in patients’ CTCs and primary tumor biopsies (Figure 3c) [36,50]. Tan et al. tracked rearrangement patterns in an NSCLC patient receiving TKI treatment and determined on-track tumoral response 3 months post-therapy with 50% decrease in ALK+ CTC counts, and CT scans revealing a shrinking tumor [36]. 2 months later, after CT scans revealed disease recurrence, a processed liquid biopsy revealed CTCs with differing ALK rearrangements, suggesting a noninvasive monitor of therapeutic efficacy.

Local and metastatic breast cancer treatment is often guided by human epidermal growth factor receptor 2 (HER2) status from the primary tumor. However, HER2 status in metastatic sites is often discovered to be different from that in the primary tumor, reducing the efficacy of treatment for the metastatic subset [51]. Identification of HER2 status in isolated CTCs could dictate biomarker targeting therapy for metastatic sites. Incongruencies of CTC HER2 expression have been investigated in HER2+ and HER2- breast cancer patients [52,53,54]. CTCs from HER2+ metastatic breast cancer patients, purified using a slanted spiral microchannel, were revealed to be HER2- whereas the primary tumor was HER2+, suggesting that continued therapy against HER2 would not be as effective [53,54]. Jordan et al. observed that cells from CTC cultures and mammary xenografts dynamically convert between HER2+ and HER2- subpopulations within four cell doublings [55]. Combinatorial therapy simultaneously targeting both populations was shown to significantly repressed tumorigenesis in mice, showing the effectiveness of complementary screening to account for the interconversion. Medford et al. detected HER2 mutations from patient ctDNA and used an ex vivo CTC culture to validate their existence [56]. CTC cultures established from two patients with known HER2 status were receptive to appropriate therapies, and change in HER2 status was verified with immunoblotting before and after therapeutic exposure.

Real-time, on/off transitions in AR-related gene expression from prostate cancer CTCs have been postulated to reflect the patient response to therapeutics and drive treatment options [29,32,52,57]. Viable CTCs from a mCRPC who had progressed through several treatment options, including androgen deprivation therapy, were isolated with the CTC-iChip and stained for androgen-driven protein prostate-specific antigen (PSA) and androgen-repressed protein prostate-specific membrane antigen (PSMA) [29]. Collectively, 2/15 CTCs were categorized as “AR-on” (PSA+/PSMA-), 2/15 CTCs were “AR-off” (PSA-/PSMA+), and 10/15 CTCs were “AR-mixed” (PSA+/PSMA+). Miyamoto et al. elucidated AR gene mutations and splicing variants within prostate cancer CTCs to assess heterogeneity across patients and among primary tumor samples [57]. In addition, from patients undergoing enzalutamide AR inhibition therapy, activation of noncanonical Wnt signaling from CTCs was linked to reduced drug efficacy. AR gene markers were also detected through RNA-Seq and ddPCR analysis of prostate cancer CTCs taken from stabilized whole blood days after CTC-iChip sample extraction [58]. Real-time determination of these profiles in response to treatment informs clinicians of appropriate therapeutics that may be effective against patient tumors.

IM-enabled CTC purification provides CTC populations with adequate purity for downstream nucleic acid analysis. Wang et al. utilized a double spiral microchannel to identify CK-19 positive CTC samples using loop-mediated isothermal amplification (LAMP) to target CK-19 mRNA, an epithelial marker [59]. LAMP-mediated detection rapidly confirmed CK-19 mRNA (40 min), providing a positive signal for samples with >33 CTCs/mL blood. In one case study, the deficiency of CK-19 in post-therapy liquid biopsies was consistent with a CT scan of the patient’s tumor, indicating successful disease treatment. Kidess-Sigal et al. found complementary clinical benefits in sequencing both colorectal cancer CTC and ctDNA to understand tumor heterogeneity [37]. Analysis of periodic liquid biopsies revealed elevated KRAS+ and PIK3CA+ ctDNA and CTC counts months after patient treatment (Figure 3d). This finding suggested drug resistance and provided evidence of disease recurrence before tumor growth was visible in CT scans. Sensitive CTC RNA expression profiling techniques demonstrate the clinical significance of heterogenetic molecular signatures in drug resistance. Drapkin et al. used the CTC-iChip to isolate CTCs from small cell lung cancer (SCLC) patients and generated patient-derived xenografts (PDX) in mice [60]. Genomic and transcriptomic analysis of early passage CTC-PDX displayed known SCLC markers and patient-specific somatic alterations. PDX were used to model patients undergoing chemotherapy and prompted the discovery of transcriptional profiles related to chemotherapy response. These studies suggest that IM isolate CTCs with preserved mutational profiles, which is necessary to monitor patients’ therapeutic response and guide treatment strategies.

CTC Biomarker Exploration

**Figure 4:**
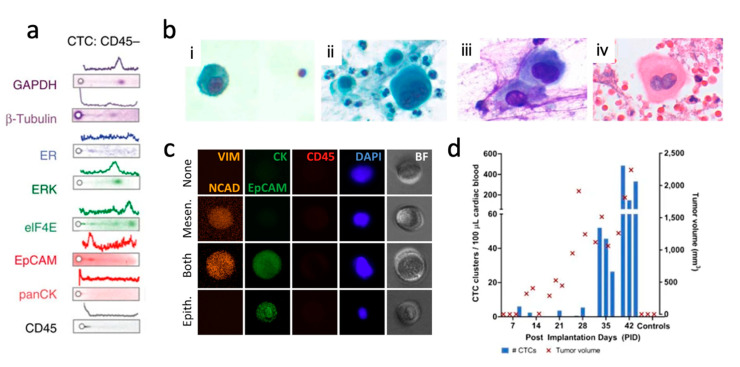
Label-free CTC isolation is necessary for unbiased CTCs studies. (**a**) Patient-derived single-CTC lysate is analyzed by single-cell Western blotting and rounds of immunoprobing against a wide array of signaling and onco-proteins relevant to cancer. Reprinted from [61]; permission conveyed through CC BY 4.0. (**b**) The cytological analysis revealed that CTC (i) revealed binucleation, which was also seen in a Pap stain (ii) and MGG stain (iii) of the patient’s pleural fluid and H&E stain (iv) of a primary tumor sample. Adapted from [50]; permission conveyed through CC BY 4.0. (**c**) Immunofluorescence staining of mesenchymal (VIM, NCAD) and epithelial (CK, EpCAM) markers revealed cellular heterogeneity. CTCs (CD45-) may or may not express these markers, depending on their EMT status. Reprinted from [68]; permission conveyed through CC BY 3.0. (**d**) Relationship between tumor volume with elevated CTC clusters counts per 100 μL of cardiac blood in a xenograft mouse model. Reprinted from [69]; permission conveyed through CC BY 4.0.

Characterizing protein expression of CTCs will help discover new biomarkers to understand cancer and select better treatments. Fachin et al. performed mass cytometry on CTCs from mCRPC patients to obtain expression levels for a wide range of protein markers, demonstrating heterogenetic profiles of CTCs in metastatic cancer [52]. Using a Vortex chip, Sinkala et al. isolated breast cancer CTCs and performed single-cell Western blotting to classify cells based on cell proliferation biomarker expression [61]. The group identified single-cell expression of HER2, epithelial cell adhesion molecule (EpCAM), estrogen receptor, and several other cancer-relevant protein biomarkers that can signal improved therapeutic choices (Figure 4a). Abouleila et al. performed single-cell mass spectroscopy on colorectal and gastric cancer CTCs to identify distinct metabolic information that is not readily conveyed from genetic or proteomic studies [62]. Interpreting these findings will help clinicians better identify CTCs and improve the efficacy of liquid biopsies in making therapeutic decisions.

In addition to protein biomarkers, CTCs isolated with IM are amenable for sequencing and identifying genomic cancer biomarkers. By performing next-generation sequencing (NGS) on Vortex-isolated CTCs, Liu et al. identified unique cancer-specific mutations, such as ATM and MSH2, present in both colorectal cancer patients’ liver metastasis biopsy and individual CTCs [63]. Yin et al. conducted single-cell whole-exome sequencing on three CTCs isolated from HER2+ metastatic breast cancer patients with the ClearCell FX platform [64]. The genotypes of three CTCs from a single patient were quite heterogeneous, as only ~5% of mutations were shared between two CTCs, and 2% were shared by all three. Winter et al. validated an integrated protocol to probe colorectal cancer CTC RNA for tumor marker discovery using a slanted spiral microchannel for cell enrichment followed by ddPCR for detecting colorectal cancer genes within enriched CTC samples [65]. Consistent mutations between collected CTCs processed with these methods may reveal new biomarkers relevant to tumorigenesis and metastasis.

Label-free isolation techniques that maintain cellular integrity for morphological studies can be used to uncover commonalities between single CTCs and primary tumors. Ozkumur et al. performed Pap smear staining on CTCs purified using CTC-iChip for clinical cytopathological analysis at standards comparable with those obtained from primary tumor tissue samples [29]. The combination of Pap smears and immunocytochemistry analysis verified oncogene aberration at both morphological and immunohistochemical levels. Dhar et al. reported that immunohistochemistry staining on CTCs were comparable to those on solid tumor Pap smears, as synonymous morphological features were detailed in both stains (Figure 4b) [50]. Similar morphologies between tumor biopsies and CTCs have also been identified in hematoxylin and eosin (H&E) staining [66].

Intact, isolated cancer cells have shown different levels of deformation compared to background blood cells, suggesting a novel biomarker that can be screened in a high throughput manner. Che et al. used a hybrid Vortex chip for atypical cell isolation and hydrodynamic compression, revealing increased deformability and larger cell size in CTCs compared to WBC controls [67]. Sample purification and CTC quantification were completed within 1 hour, compared to the standard 3+ hours required for off-chip immunostaining. This methodology identified more NSCLC patients with CTCs (93.8%) and, thus, elevated metastatic potential compared to traditional immunostaining methods (71.4%).

Investigations into CTC Biology

The label-free nature of IM allows for an unbiased collection of atypical CTCs regardless of surface profiles, enabling comprehensive surface marker heterogeneity profiling. Following enrichment, several groups have screened candidate CTCs for epithelial markers (i.e., CK, EpCAM), mesenchymal markers (i.e., VIM, NCAD), and DAPI staining to better understand CTC subpopulations (Figure 4c) [44,68]. Zeinali et al. used a Labyrinth chip for both CTC and CTC cluster isolation, and off-chip staining revealed that 17/23 NSCLC patients exhibited more EpCAM- CTCs than EpCAM+ CTCs [44]. Amongst all captured CTCs, 45% of cells expressed Vimentin, a mesenchymal marker, emphasizing the heterogeneity of CTC samples and the need for more stringent characterization. Several studies have used IM to identify EpCAM- CTC populations, which a subpopulation of CTCs undergoing the epithelial to mesenchymal transition (EMT) [68,70]. Although the percentage of CTCs undergoing EMT varies drastically due to patient heterogeneity and different disease stages, identification of EMT+ fractions of CTC may provide new insight in assessing tumor progression. PDX models of triple-negative breast cancer (TNBC) generated from Vortex isolated CTCs revealed heterogeneous CTC populations within the mice [69]. In 1/7 models that were aggressively metastatic, the majority of CTCs were undergoing EMT changes, revealing the need for further studies to correlate EMT status and metastasis. Heterogenetic EMT status within CTC population suggests high throughput and accurate screening of CTCs is paramount to identify cells capable of initiating the metastatic cascade.

IM devices have also shown the ability to isolate variably sized CTC clusters that may be a key contributor in cancer metastasis [71]. Vortex-isolated CTCs from triple negative breast cancer (TNBC) patients were used to generate PDX models The PDX TNBC model showed that the progression of metastasis and tumor burden was correlated with elevated CTC counts and clusters from collected blood samples (Figure 4d) [69]. In a survey of 60 head and neck cancer patients of varying stages, CTC clusters were found in all 15 stage IV patients. [72]. Within their cohort, cancers did not metastasize in 95% of patients without CTC clusters, suggesting a target for further investigation. Other studies also have shown the increasing number of CTC clusters in HCC and head and neck cancer patients with more advanced stages of cancer [30,73].

IM allows for the studying of the mechanism of metastasis because CTCs purified using such systems preserve their cellular activity and transient phenotype [74]. There is merit in identifying the fractional subset of CTCs with the capacity to metastasize to better understand the inefficient process [75]. Zheng et al. postulated that oxidative stress programmed CTCs to adopt survival mechanisms in otherwise harmful environments [74]. CTCs were isolated with the CTC-iChip, cultured, and inoculated into immunosuppressed mice for in vivo tumor growth. Hydrogen peroxide-induced reactive oxygen species (ROS) activates β-globin, which allows CTCs to resist ROS-mediated cytotoxicity and continue circulating throughout the system. Dhar et al. developed a hybrid Vortex chip capable of encapsulating purified CTCs in droplets to measure their single-cell level MMP activity, which has been strongly implicated in orchestrating tumor cell invasion [75]. Ebright et al. found that RPL15, a ribosomal subunit, was overexpressed in mouse models used to study distant metastasis [76]. CTC-iChip CTC isolation and downstream RNA sequencing validated this finding in patient samples, as samples overexpressing RPL15 had other markers related to worse progression-free survival.

CTCs have been posited to have stem cell-like qualities, which aid metastasis and tumor development [30,70]. Lin et al. classified breast cancer CTCs enriched using a Labyrinth chip into 4 distinct subpopulations indicative of tumor stem cell status by performing single-cell quantitative real-time polymerase chain reaction (RT-PCR) [70]. Aya-Bonilla et al. used a slanted spiral microchannel to isolate melanoma CTC fractions for secondary processing [77]. Multimarker flow cytometry and RT-PCR conducted on CTCs from patient samples confirmed the heterogeneity of melanoma CTCs as few selected markers were broadly expressed. These methods revealed the expression of stem-cell-like genes ABCB5 and PAX3 in 6/7 patients with metastatic cancers, supporting the idea that these genes are critical for disease progression. Wan et al. isolated HCC CTCs and found increased expression of CD44 in more advanced stage patients, also indicative of increased cell stemness [30]. Beyond aiding with therapeutic selection, studying IM-isolated CTCs is useful to better understand the underlying mechanisms of CTCs.

Summary and Outlook

In the past few years, IM has improved our understanding of CTC biology with clinical benefits. In contrast to the FDA-approved affinity-based methods, IM is a label-free CTC isolation method inclusive of CTC genomic and proteomic heterogeneity. Furthermore, IM provides gentle CTC isolation, which is critical for performing secondary assays on viable captured cells. Isolated CTCs have been used most prominently to investigate protein expression, genetic makeup, and patient response to therapeutics. Collectively, these studies improve our understanding of genomic and proteomic interpatient and intrapatient heterogeneity. With this knowledge, clinicians can make more effective therapeutic decisions to improve cancer patient health.

As a relatively nascent field, new channel geometries are continuously developed to further improve device performance. For example, Mishra et al. further modified the CTC-iChip by adding a strong magnetic sorter to improve throughput 100-fold and isolate CTCs from concentrated leukapheresis products [78]. Edd et al. focused on gentler CTC cluster isolation by using angled rectangular islands for inertial focusing and repetitive flow shifting [79]. The addition of inline trapezoidal or downstream DLD pillars to spiral channels was shown to improve sample purity and separation efficiency, respectively [80,81]. Xiang et al. proposed a different device with 3 spiral microchannels in parallel, connected with cross channels, to simultaneously isolate CTCs and concentrate diluted cells [82]. Gao et al. combined contraction-expansion arrays with an asymmetric curved and bifurcated channels to further improve isolation purity and capture efficiency [83]. Others have added siphoning channels in CEA chambers for continuous cell sorting [84,85]. Using a negative selection strategy, Lee et al. reported a curved and contraction-expansion chip that used IM to thoroughly mix WBC-binding beads in samples prior to CTC isolation [86]. Straight channels with the additional co-flow of buffer or viscoelastic fluid have also shown promise for label-free CTC isolation [87,88].

Future work should be dedicated to diversifying the types of cancer and patient samples investigated. The majority of current IM applications for CTC purification has focused on lung, breast, and prostate cancer CTCs. Increased proteomic and genomic studies of other cancers will improve our understanding of CTC biology and the promising role that CTC profiles can play in the clinic. This should be enabled by increased adoption of IM to study cancer cells in various types of bodily sample, including blood, pleural effusions, and urine. More in-depth studies should be conducted on CTC clusters, which are related to increased metastatic potential [71]. Accessibility to IM technologies is rapidly improving with the commercialization of both the Vortex chip and ClearCell FX platform. The development of these companies illustrates the immense promise of IM in studying cancer.

### 2.2. White Blood Cells

Introduction

Granulocytes, lymphocytes, monocytes, and other white blood cells (WBCs) play a vital role in generating an immune response to different diseases [89]. In-depth investigation of these cells, including differential cell count and activation state profiling, would provide a wealth of information about patient health status for a variety of diseases, such as sepsis, diabetes, and cancer [90,91,92,93]. To perform such analyses, WBCs must first be purified from blood samples, but existing affinity- and density-based WBC separation methods are both time and resource-consuming, leading to WBC activation state alteration; thus, obfuscating valuable information pertinent to patient state [94,95]. Inertial microfluidics (IM) addresses these shortcomings with hydrodynamically driven separation of WBCs from other blood components without exposing cells to biomolecules or forces that may alter their viability or activation state. Successful analysis of IM isolates has validated the efficacy of IM in purifying WBCs from heterogeneous samples while preserving activation states. Recent studies have demonstrated applications of IM towards characterizing a host of diseases by characterizing WBCs. A summary of all devices reported in this section is in Table 1.

Respiratory Illness

Respiratory illnesses, such as chronic airway disease and accumulation of pleural effusion, affect lung function and compromise patients’ health and quality of life. To better understand an individual’s respiratory condition, WBCs can be collected from patient samples, such as pleural fluids and airway secretions, to determine the severity and cause of the illness [104,105,106,107]. However, current techniques for purification and subsequent analysis of WBCs are time-consuming, compromise sample integrity, or require additional testing to provide accurate assessments. IM provides an alternative method that passively purifies WBCs without compromising sample integrity.

**Figure 5:**
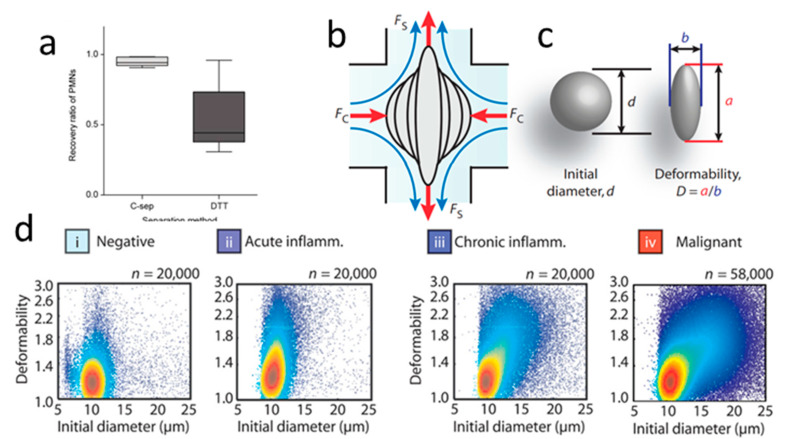
Separation and assaying of WBCs, from patients with respiratory illnesses. (**a**) The separation efficiency of the inertial microfluidic device (left) vs. traditional Sputalysin coupled centrifugation (right). Panel reprinted with permission from [95], Copyright 2017 American Chemical Society. (**b**) Schematic of how cell deformation occurs in inertial focusing and (**c**) measurements before and after deformation. (**d**) Sample output of the device, plotting size versus deformability, and representative plots of a negative (i) diagnosis, acute inflammation (ii), chronic inflammation (iii), and detection of malignant cells (iv). Panels b-d reprinted from [114], with permission from AAAS.

Chronic airway diseases, such as cystic fibrosis (CF) and chronic obstructive pulmonary disease (COPD), affect approximately 12 million patients in the US [108]. In acute cases, information-rich neutrophils infiltrate patients’ airways, enabling non-invasive cell collection from sputum [104,107,109,110]. There is high sputum viscosity variability between patients, necessitating chemical homogenization techniques that alter transient WBC activation state, resulting in inaccurate surface marker interrogations [111,112]. As a gentler alternative, large volume dilutions can be used to transform viscous polymeric sputum into processable consistency [95,113]. IM is uniquely suited to process large volumes of diluted sputum solution in a high throughput manner to sift infiltrated neutrophils out from very dilute sputum [2]. Ryu et al. processed 50 mL of a 1000× diluted sputum sample through a spiral microchannel in 13 minutes with a high neutrophil separation efficiency (95%), an improvement over the conventional method (53.5%) (Figure 5a) [95]. Neutrophil elastase activity analysis on all samples homogenized by dilution showed reduced artificial activation.

Congenital heart failure, cirrhosis, tuberculosis, and other bodily diseases generate pleural effusions (PEs), the excess fluid buildup in the lining of the lungs [115]. Malignant pleural effusions (MPEs) describe particularly dire cases where malignant cancerous cells infiltrate the pleura, occupying 4–12% of the approximately 1.7 × 10^3^ WBCs/mL in PEs [116]. Fluorescent labeling techniques have been used to differentiate cells found in PEs and determine the underlying illness, but these methods are hampered by high false-positive rates and lengthy processing times [114]. Gossett et al. and Tse et al. developed a method for IM-assisted deformability interrogation and interrogated infiltrating WBCs in PEs as an attractive alternative for rapid assessment of patient status [103,114]. Cells collected from PEs were hydrodynamically deformed in a high throughput manner, and populations were plotted based on deformability and initial cell size at single-cell resolution (Figure 5b,c). Upstream inertial focusing with an asymmetrically curved channel ensured high accuracy by standardizing cell position prior to deformation and minimizing interference from other cells during deformation. PE samples could be classified based on deformability trends, prompting further patient testing for acute/chronic inflammation and MPEs (Figure 5d). Data collected from this device identified fractions of non-WBC infiltrating cells in PE samples and helped estimate the likelihood of the sample containing metastatic cancer cells. Amongst 116 patients with PEs, Tse et al. reported that the deformability analysis properly screened 56 (47%) patients as negative and 19 (16%) patients who were positive for malignant cells. Integration of this device into the sample analysis pipeline could reduce the number of false positives and the need for follow-up screenings [103].

Diabetes

Diabetes mellitus (DM) remains a serious worldwide public health challenge, with a forecasted 360 million cases by 2030 [117]. Patients with type 2 diabetes mellitus (T2DM) are often at risk for cardiovascular diseases. Looking for markers of T2DM and cardiovascular diseases in DM patients would enable physicians to identify patients at risk for either condition and to monitor and treat them accordingly. The risk of cardiovascular disease and inflammation in DM patients can be assessed by profiling neutrophil phenotypes and the tendency to form neutrophil extracellular traps (NETosis) [118].

**Figure 6:**
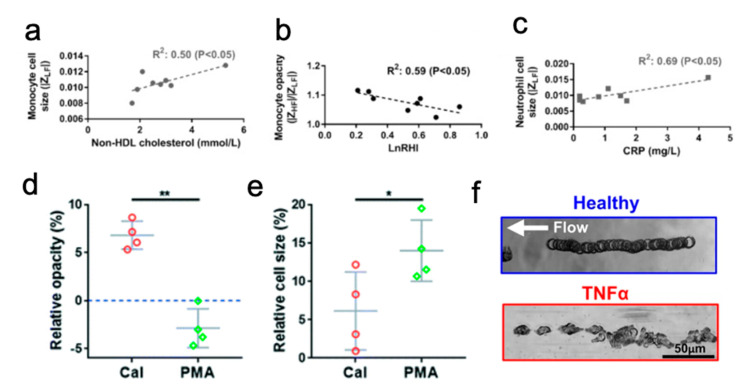
Interpretation of results from impedance measurements (**a**–**c**). Correlations between (**a**) monocyte cell size and cell lipid content, (**b**) monocyte opacity and vascular function as measured by logarithm of reactive hyperemia index (lnRHI), (**c**) neutrophil size and CRP levels. Panels a-c reprinted with permission from [96], Copyright 2018 with permission from Elsivier. Changes in neutrophil (**d**) opacity, and (**e**) size when treated with calcium ionophores (CaI) and phorbol 12-myristate 13-acetate (PMA) to stimulate NETosis. Both figures are normalized to a control of unstimulated neutrophils. Panels d-e reprinted used with permission of MDPI, from [97]; permission conveyed through Copyright Clearance Center, Inc. (**f**) Time lapse captures showing the difference between a healthy rolling profile (top) and the discontinuous cell “flips” when treated with TNF-α (bottom), panel used with permission of Nature Publishing Group, from [99]; permission conveyed through CC BY 4.0.

Hou et al. integrated a spiral microchannel with impedance cytometry for leukocyte sorting and electrical profiling using diluted or RBC-lysed blood samples [96,97]. Larger neutrophils and monocytes (~10–12 μm) were focused closer to the inner wall of a spiral channel with 90% purity. Cell size and electrical opacity were profiled with inline impedance gating at different frequencies. Monocyte opacity readings and neutrophil size both correlated with increased cardiovascular risk (Figure 6a–c). Changes in cell size and opacity were also observed in neutrophils undergoing NETosis, a defense immune response to inflammation leading to tissue damage in diabetes, when compared to unstimulated groups (Figure 6d,e) [97].

Neutrophil behavior was then studied in healthy donors vs. T2DM patients by implementing a microfluidic chemotaxis assay [98]. Tay et al. found that when loading the chamber with the chemoattractant *N*-Formylmethionyl-leucyl-phenylalanine, IM purified activated neutrophils from TD2M patients had a much slower chemotaxis speed than inactivated neutrophils from healthy donors. Furthermore, comparative chemotaxis velocities pre-and post-treatment of cells in vitro with the anti-diabetic drug metformin showed a significant increase in neutrophil chemotaxis speeds for some patients [98]. These findings suggest IM purification and subsequent chemotaxis assays as simple tests for assessing patient risk and subsequent therapeutic options.

Label-free neutrophil purification using IM enables the subsequent use of rolling assays to diagnose and monitor T2DM, as the isolation technique preserves surface profiles. IM-purified neutrophils treated with glucose rolled straight on an E-selectin coated microchannel under physiological shear conditions. Curiously, neutrophils treated with tumor necrosis factor alpha (TNF-α), a neutrophil activator that mimics T2DM conditions, moved rapidly in a flipping motion in the same [99]. Visualizing this stark contrast in behavior serves as a potential assay to quickly characterize whether a T2DM status (Figure 6f).

Sepsis

Sepsis is an acute, systemic response to an infection, where a rapid clinical response is critical because every passing hour decreases the probability of patient survival by 7% [119]. In response, broad-spectrum antibiotics are typically administered to quell the infection at the expense of potentially generating antimicrobial-resistant (AMR) bacteria [120]. A robust, rapid test that requires minimal sample preprocessing that can determine whether a patient is septic would allow physicians to correctly prescribe antibiotics, reducing the risk of misdiagnosis and overprescription.

Using a curved channel to align WBCs in a channel and control cell speed, Crawford et al. employed a hydrodynamic deformability cytometer (Figure 5b,c) to identify a physical biomarker indicative of sepsis within 10 minutes of drawing blood, possibly preventing incorrect administration of antibiotics [102,103]. In general, granulocytes in septic patients were more deformable than that in their healthy counterparts. The group identified a threshold for granulocyte deformability that indicates sepsis with 96% sensitivity and 100% specificity [102]. IM processing is well suited to diagnose an illness as time-sensitive as sepsis as the required high flow rate permits rapid physical analysis of individual cells to draw quick conclusions.

**Figure 7:**
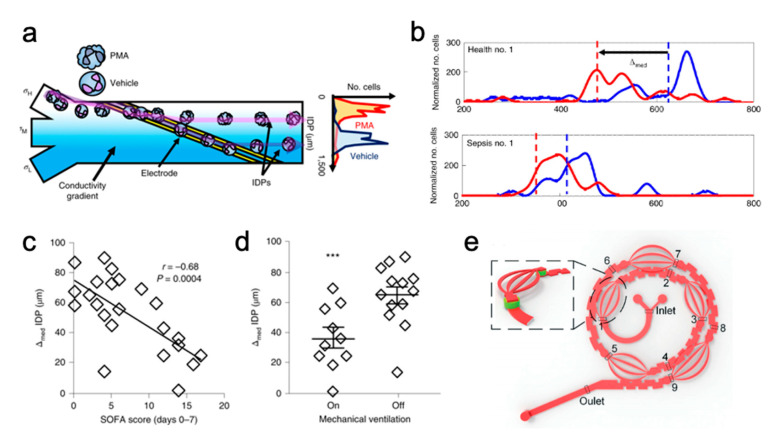
Downstream isodielectric point (IDP) assay, and 3-D geometry for RBC lysis. (**a**) Illustration of the microfluidic channel used to determine the isodielectric point), as well as peak locations for cells treated with an activating agent versus just the drug carrier. (**b**) Example of Δ_med_ calculations between healthy donors (top) and septic patients (bottom). The focusing positions shown are of PMA activated cells (red) vs. cells exposed to the vehicle (blue). Plots showing the correlation (**c**) between the Δ _med_ and SOFA scores over 7 days of patient treatment (**d**) between the ∆_med_ and the need for mechanical ventilation. Panels a – d reprinted with permission from [94], Copyright 2019 Springer nature. (**e**) Schematic of the lysis module showing the 3-dimensional channel from the inertial microfluidic cube used for mixing the lysing agent and whole blood. The enlarged section shows the 3-D flow path, panel used with permission of Royal Society of Chemistry, from [100]; permission conveyed through Copyright Clearance Center, Inc.

Jundi et al. used a spiral microchannel to isolate granulocytes from peripheral blood to look for biomarkers indicative of sepsis and cell activation [94]. Expression levels of activation markers, such as CD11b, CD66b, and CD18, were preserved after IM separation but not after centrifugation [121,122,123]. These isolated granulocytes were further analyzed to discover potential sepsis biomarkers, such as lower levels of CD16 expression (40% reduction), neutrophil-elastase release (63% reduction), and O_2_^−^ production (90% reduction) compared to non-septic blood samples. Lastly, differences in isodielectric focusing positions pre-and post-activation were correlated with sepsis severity (Figure 7a,b) [94]. IM can enable the routine use of isodielectric position assays to routinely monitor patient status and give physicians feedback on patient treatment because it can quickly, automatedly, and efficiently separate granulocytes from microliter quantities of blood.

WBC Isolation with Hemolysis

Hemolysis is a necessary step for many assays to minimize background signals and enhance collected target purity [99,102]. On-chip hemolysis substantially shortens the analysis process and reduces background noise for subsequent assays [102,103]. Zhu et al. developed a fully integrated 3-D IM device that lyses RBCs and purifies leukocytes from undiluted blood (Figure 7e) [100]. Fabrication was done using laser-cut polymer sheets bound together with double-sided tape. They reported a separation efficiency of 84%, with high cell viability of 96.6%, showing minimal lysis of WBCs. Ramachandraiah et al. developed an IM device with 2-D curved channels and serial outlets, capable of osmotic hemolysis on the chip, to fractionate granulocytes, monocytes, and lymphocytes based on their sizes [101]. The group reported a 98% WBC separation efficiency of blood with 5% dilution, showing that the lysis buffer had little impact on the WBCs.

Summary and Outlook

IM systems showcased in this section suggest that IM provides an effective alternative to the current gold standard of WBC purification and fractionation. The label-free purification that IM provides enables collecting WBCs with preserved integrity, thus improving the accuracy of downstream testing, allowing for the discovery of new physical and electrical biomarkers, and exploring additional biological assessments [99]. These technologies have shown promise in making the transition from benchtop to the clinic. The deformability device mentioned in this section has been commercialized by Cytovale, with the eventual goal of integrating the device into the clinical workflow.

### 2.3. Reproductive Health Related Targets

Introduction

IM devices have recently been employed in reproductive health as automated, label-free purification tools for sperm cells and fetal trophoblasts. The collected clinical specimens are often contaminated with high background levels of blood cells that must be removed, but common affinity-based separation methods are time-consuming and labor-intensive, and in some cases, the associated collection methods risk the patient’s health [124,125]. Due to a large size difference between reproductive cells and blood cells, IM can play a niche role in efficiently separating these cells of interest and provide high-purity samples for analysis.

Purification of Reproductive Health Related Cells

**Figure 8:**
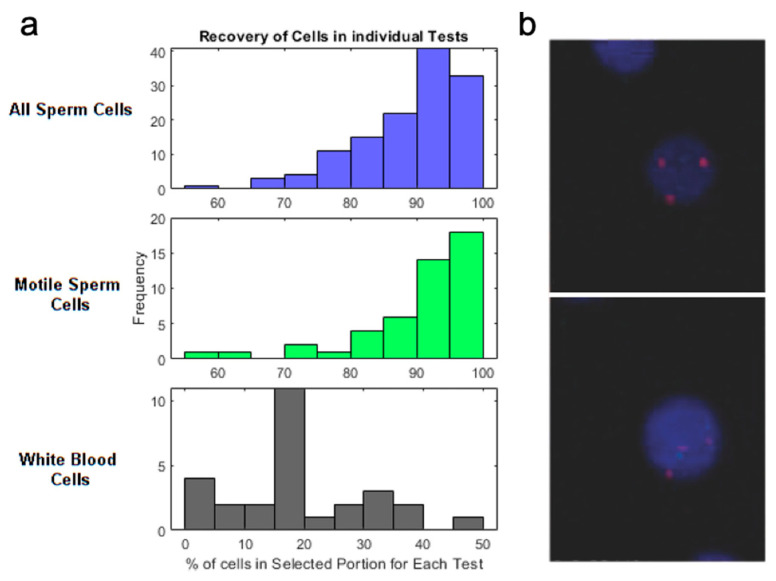
IM separation of reproductive health targets. (**a**) Histograms of recovery percentages for all sperm cells (top), motile sperm cells (middle), and WBCs contaminants (bottom). Reprinted by permission from [138]. Copyright 2020, Springer Nature. (**b**) FISH staining of purified trophoblasts for trisomy 21 on chromosome 21 (red, top) and chromosome 13 (green, bottom). Adapted from [143], © 2018 WILEY-VCH Verlag GmbH & Co. KGaA, Weinheim.

Assistive reproductive technologies (ART) is a class of therapies used to treat infertility. ART used to treat male infertility requires the accurate assessment of sperm, WBC, and RBC counts in seminal fluid extracted via a surgical procedure, testicular sperm extraction (TESE) [126,127]. Previously, microfluidic devices relied on sperm motility for separation, often discarding viable sperms with low motility, thus leading to inaccurate assessment of viable sperm counts in the sample [128]. Son et al. used a spiral microchannel to purify viable sperms from seminal fluids and achieved 80% recovery of sperm while removing 92% and 87% of WBCs and RBCs, respectively, using donor sperm samples spiked with blood cells to replicate pyospermia (high WBC semen) [129,130,131,132]. Overall, IM isolation of sperm demonstrated an almost nine-fold increase in sperm recovery than other conventional methods (e.g., centrifugation, manual sorting) and an 81% increase in the capture of non-motile sperm compared with non-inertial microfluidic sperm isolation systems [133,134,135,136]. Additionally, unaltered viability observed in sperms 30 min after the process suggests the possibility of reprocessing samples for further enhancement of purification and enrichment [134,137]. By separating both motile and non-motile viable sperms with high efficiency and purity, these IM devices showed promise for automatable sample preparation and the possibility to incorporate downstream assays, such as sperm motility assay. Finally, the group optimized a sperm separation protocol to be used with intrauterine insemination. Compatibility of isolated sperm with intrauterine insemination is assessed using the three criteria: injection volume less than 1 mL, no WBCs, and removal from seminal plasma [136]. Jafek et al. developed a pipeline consisting of a single centrifuge wash and subsequent run through a microfluidic chip, showing an average recovery rate of 90% across 130 tests (Figure 8a, top) of the motile sperm, and meeting all the criteria for intrauterine insemination [138]. The purified sample only retains on average 18% (Figure 8a, bottom) of WBCs and is washed of seminal plasma, with the final volume < 1 mL, showing promise for intrauterine insemination.

Diagnostic tests to confirm chromosomal abnormalities require collecting samples of amniotic fluids or placenta. These invasive tests increase the risk of miscarriage and deformation of the fetus [125]. Isolating fetal analytes circulating in maternal blood, including cell-free DNA (cfDNA) or fetal trophoblasts (CFTs), could provide a minimally invasive alternative screening method [139]. CFTs are rare cells in maternal blood (1–5 trophoblasts per mL) that can provide information on genetic defects such as aneuploidies, unbalanced translocations, sub-chromosomal deletions, and duplications [140,141]. Utilizing their large diameters (>15 μm) [142], Winter et al. used a micro-milled slanted inertial microfluidic device to enrich trophoblastic cells with a one-minute processing time [143]. The group successfully separated CFTs from 7 mL of lysed maternal blood from a patient who has a high risk of having a baby with Down syndrome with 99.5% depletion of WBC and 75% of trophoblasts recovery within a few minutes [143]. The group then confirmed the presence of the genetic mutation, trisomy 21, by FISH staining (Figure 8b). Lastly, the feasibility of prenatal diagnosis was tested with single-cell sequencing from model XY-JEG3 cells purified from whole blood, demonstrating high enough purity for sequencing. While awaiting further clinical validations, IM trophoblast isolation approaches could provide prenatal health testing with minimal risk.

Summary and Outlook

For both sperm cell isolation and high-risk pregnancy testing, size-based isolation can establish a new gold standard. Using IM to separate the smaller sperm cells from blood cell contaminants, clinicians can obtain accurate viable sperm counts. Inertial separation of fetal trophoblasts presents geneticists with an alternative to obtaining vital fetal information from a non-invasive maternal blood draw [125,143]. These two applications show another field where inertial microfluidics can simplify the workflow and help clinicians treat patients.

### 2.4. Plasma

Introduction

Plasma is a straw-yellow liquid that makes up 55% of the blood volume. Accurate and reproducible measurement of analytes in plasma such as proteins, ions, metabolites, and nucleic acids is critical for the diagnosis and prognosis of various diseases [133,144], including cancer [134], diabetes [135], autoimmune disease, and malaria infection [136]. Complete, timely depletion of cellular components after blood collection is crucial for plasma extraction and analysis because proteins and nucleic acids from degraded cells (i.e., hemolysis and leukolysis) can interfere with the downstream analysis [133].

Centrifugation, the “gold standard” for plasma extraction, yields high-purity (~100%) plasma [137]. However, centrifugation requires a skilled technician and is labor-intensive, time-consuming, and prone to contamination [144,145]. The high mechanical stress that cells experience during centrifugation may cause inaccurate outcomes in analyte measurement [146,147,148]. Therefore, the microfluidics community has been seeking new methods of extracting plasma [149]. Inertial microfluidics (IM) promises a compact, affordable device with timely extraction and small sample consumption. Seamless integration with downstream analysis would warranty automated, reproducible analysis and reduced exposure of potentially hazardous samples to the user and environment [133,144,149]. Various microfluidic plasma extraction methods based on sedimentation, filtration, or DLD have been demonstrated [133,144]. Compared to these methods, IM plasma extraction offers simpler fabrication, less clogging, and higher throughput in a continuous flow-through manner [150].

Recent Advances in Inertial Microfluidic Plasma Extraction

Ideally, there is a single equilibrium position of cell streams for effective plasma extraction [151,152,153]. However, particles with different sizes tend to focus at different positions in a straight channel with a rectangular cross-section because inertial lift forces strongly depends on the size. Since forming a single focusing-stream of polydisperse particles like blood cells (RBCs, WBCs, platelets) in a simple straight channel is challenging [153], micro features, such as rectangular cavities or slanted grooves, were used to induce secondary flow (i.e., Dean flow) to create single focusing stream [137,148,154,155]. Curved channels, such as serpentine and spiral microchannels, are also effective for such a purpose. Dean drag forces can accelerate the focusing process [153], so the spiral microchannel design benefits from a smaller device footprint. Various channel cross-sections (e.g., low-aspect-ratio rectangular and trapezoidal) and various microfeatures (e.g., micropillar filters, microbar obstacles) were employed to further enhance plasma separation [151,152,153,156,157,158].

For a straight-channel device with microfeatures, Lee et al. exploited a contraction-expansion array (CEA, horizontal arrangement) and sheath flow [148,155]. Although inevitable dilution of plasma due to the high flow rate of sheath flow used to confine plasma into a narrow stream, a remarkable 69.5% plasma yield was obtained. Zhao et al. constructed a vertical arrangement of a sheath (top) and sample flows (bottom) in a double-layered microchannel to implement 3-dimensional slanted grooves in a conventional horizontal arrangement [154]. The sheath flow and transversal secondary flow induced by the grooves helped to confine cells into a narrow stream. Although microfabrication of the two-layered structure could be challenging compared to other single-layer microfluidic devices [148], an impressive 99.9% purity was achieved using undiluted blood. 

**Figure 9:**
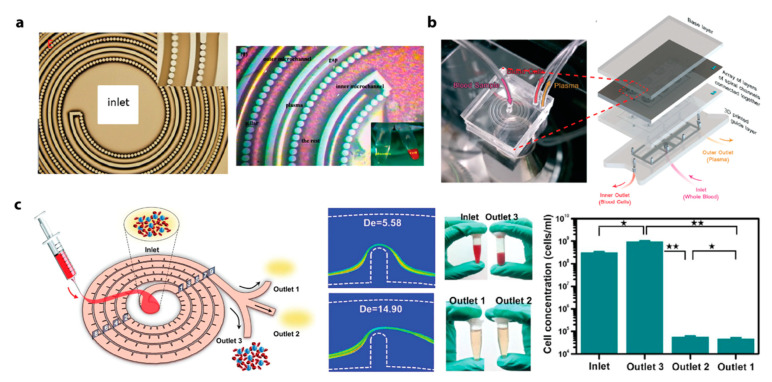
Inertial microfluidic devices for plasma extraction. (**a**) Micropillar filters in a spiral microchannel were used to separate cells from diluted blood. Separated plasma and concentrated blood cells are shown in the silicon microfluidic device and microcentrifuge tubes (inset figure). Adapted from [156] with permission from the author, Copyright 2013, Elsevier. (**b**) A spiral microfluidic device with the trapezoidal cross-section was effective in focusing diluted blood cells into a single stream for plasma extraction. The total 16 devices were parallelized for enhanced throughput. Adapted from [152] by permission from The Royal Society of Chemistry. (**c**) A spiral microchannel with regularly spaced microbars improved the focusing of a blood cell stream by secondary Dean-like flow. The optimized microbar shape and operating condition allowed higher purity than that reported in the authors’ previous work with unoptimized conditions [153]. A manual plasma extraction was demonstrated using a syringe (15x diluted blood),adapted from [157] with permission from the author, Copyright 2014, Elsevier.

Zhang et al. integrated a membrane filter to eliminate residual blood cells that were not completely removed from initial IM separation using a serpentine channel [159]. The use of the additional filter achieved nearly 100% purity (Figure 9a). To overcome a low processable volume capacity limited by the plasma chamber size and retaining filtered cells, they parallelized eight devices to achieve an impressive 2.8 mL/min throughput from diluted blood (20×). 

Xiang et al. proposed a spiral microchannel plasma extraction device with a rectangular cross-section [151]. Although the plasma yield was 38.5%, almost 100% purity was achieved from 20× diluted blood, aided by the induced secondary flow. Geng et al. reported evenly spaced micropillars filters (1.7 μm gap) in a spiral microchannel. Plasma was extracted from 20× and 12× diluted blood samples in their silicon microfluidic device (Figure 9b), fabricated using photolithography and silicon etching [156].

A spiral microchannel with a trapezoidal cross-section was adopted to utilize strong Dean vortex cores formed near the inner wall due to cross-section asymmetry [152,158]. The slanted cross-section near the inner wall helped lessen steric crowing and form a narrow particle stream (Figure 9b). Parallelization of 16 devices boosted throughput (24 mL/min) to compensate for these high dilution factors (90× and 45×) while maintaining nearly 100% purity. However, significant dilution still poses an analytical challenge for low abundant targets in plasma [133].

By combining a spiral microchannel with equally spaced microbar obstacles, blood cells were more effectively focused into a single stream [153]. Through systematic optimization process, the authors had chosen 450 μm × 200 μm microbars and π/15 angle between neighboring microbars [157] and purify plasma from 15× diluted blood with high purity (99.99%) and throughput (up to 15 mL/min, Figure 9c). This system’s ability to tolerate variations in operational flow rates enabled plasma extraction using a hand-operated syringe with 99.99% purity and 67.57% of plasma yield.

Key Design Consideration for Inertial Microfluidic Plasma Extraction

Here we briefly summarize important design aspects and device performance metrics presented in the reported studies (Table 2) to guide readers considering working on IM plasma extraction. First, the purity of plasma should be high due to the adversary effects of residual cells on analytes and clogging of microfluidic devices [144]. Plasma purity achieved via conventional centrifugation reaches almost 100% [159]. A handful of groups accomplished this level of purity using IM [151,152,159]. Towards the goal of improving purity to the centrifugation level, the papers reviewed here proposed different channel shapes (e.g., straight or curved channel; low aspect ratio rectangular or slanted cross-section), microfeatures (e.g., cavity, groove, micropillar filter, or microbar obstacle), and additional components (e.g., membrane filter).

Second, it is advantageous to use minimally or no diluted blood sample to reliably detect low-abundant analytes [147]. However, processing undiluted blood is challenging because of “steric crowding” of cells, especially RBC (~5 million cells/μL), preventing the formation of a sharp blood-cell stream and resulting in cell leakage into a plasma stream. Therefore, samples were usually 15 to 20-fold diluted before processing. The purity and the dilution factor were correlated; that is, processing higher hematocrit-content (i.e., less diluted) samples led to lower purity [149]. Plasma extraction from the undiluted blood exhibited relatively low plasma yields or required sheath flow for a proper separation [148,154]. It should be noted that sheath flow inevitably dilutes plasma and adds complexity to the extraction system. 

Third, throughput should be high enough to process more than 1 mL of whole blood in order to detect trace-quantity analytes, such as scarce proteins, peptide markers, circulating nucleic acids (CNAs), and pathogenic DNA/RNA [133]. Additionally, slow processing may lose the integrity of plasma [144]. The dilution factor should be considered in throughput evaluation because the overall sample volume will be multiplied by the same factor [152]. Therefore, we defined the throughput in this work as the time that is required to process 1 mL of an undiluted sample (Table 2): throughput = 1 mL/(flow rate/dilution factor). The reported throughput ranged from 2 min (no dilution) [154] to 2000 min (20× dilution) [156]. There exists an upper limit of throughput because a stream of separated blood cells tends to widen at higher flow rates, resulting in cell-leakage to extracted plasma (i.e., purity deterioration). Therefore, multiplexing was one approach to push the throughput beyond the limit of a single device. Up to 16× multiplexing was demonstrated to boost throughput [137,152].

Fourth, plasma yield, the ratio of the extracted plasma volume over the initial blood volume, should be high because of the large plasma quantity requirement for the detection of trace analytes. The net concentration of an analyte in the extracted plasma is smaller for the systems processing diluted blood samples than those processing undiluted samples. For example, 60% yield for undiluted blood implies a much higher analyte concentration, compared to the same yield for 20× diluted blood. Plasma yield, as listed in Table 2, varied from 38.5% to 69.5%.

Lastly, two additional design considerations are noteworthy. Mechanical stress should be minimized to prevent hemolysis and leukolysis that may interfere with downstream analysis [144,147]. In some literature, potassium [156] and hemoglobin [153] were analyzed to evaluate the hemolysis level. Simple device fabrication is preferred (e.g., single-layer PDMS device vs. multilayer PDMS or silicon device) for future commercialization [149].

Summary and Outlook

As plasma extraction is a fundamental step to undertake for the detection of valuable analytes from the blood, significant efforts have been made to build robust inertial microfluidic plasma-extraction devices. Ideally, it is desirable that the device does not depend on sheath flow, process blood in a high throughput, produce high-yield plasma for accurate and reproducible downstream biomarker analysis [157]. Additionally, the device is better to have a simple structure, have a small footprint, be inexpensive to microfabricate, and be seamlessly integrated with downstream analysis to be a viable and possibly portable alternative to the gold-standard centrifugation. 

One of the biggest challenges in IM plasma extraction was processing a high blood-cell content (~45% hematocrit level). The dilemma was that a low dilution factor (i.e., high hematocrit level) led to a low purity while a high dilution factor (i.e., low hematocrit level) or the use of sheath flow resulted in low analyte concentration in extracted plasma. Many engineering innovations, including a spiral microchannel with slanted cross-section or microbar obstacles, have been implemented to process less diluted blood and achieve higher purity. Some authors achieved an impressive near 100% purity with 15–20× dilution factors. Sheath flow also allowed the processing of undiluted blood with an excellent 70% plasma yield and 2 min throughput but resulting in inevitable plasma dilution. Even with this progress, the unmet need is unsheathed processing of undiluted blood with the level of purity and yield offered by the centrifugation. The detection of low-abundant analytes would be challenging with 15–20× diluted blood or extracted plasma aided by sheath flow [133]. However, as the inertial microfluidics field is growing at an outstanding pace, we may able to see the dream inertial microfluidic plasma-extraction device realized in the near future.

### 2.5. Extracellular Vesicles

Introduction

Extracellular vesicles (EVs) are membrane-bound vesicles that carry proteins, lipids, and nucleic acids from the host cell and play crucial roles in cellular communication and shaping of the cells’ local environment [160]. They can contribute to the progression of diseases by restricting viral replication and mediating tumor progression [160,161]. EVs are classified commonly into subpopulations of exosomes, microvesicles, and apoptotic bodies based on their diameter, with exosomes being smallest at about 100 nm. Considering the many roles played by EVs, it is paramount to accurately and efficiently collect and characterize the protein and genetic profiles of EVs. Understanding the structure and function of EV subpopulations can lead to the identification of diagnostic biomarkers and therapeutic options [160]. However, there is a general lack of methods by which EVs can be isolated and characterized accurately, as well as a large variability across EV isolation techniques [162]. The current gold standard technique for isolation, ultracentrifugation, is time-consuming, requires expensive equipment, and cannot easily separate subpopulations of EVs or contaminating proteins, limiting its use in a clinical setting [163]. IM provides a high-throughput and high-purity alternative for isolating and sorting EVs to be used in downstream clinical analyses [1,164].

Purification of Extracellular Vesicles

Affinity-based methods can be marker-specific alternatives to isolate exosomes by targeting common surface markers, such as antibody targets (CD63, CD81) or epithelial cell adhesion molecules (EpCAM) [165]. Dudani et al. designed an IM platform for isolation of microscale beads coated with antibody-captured exosomes from both cultured cells and donor blood. After the incubation period for binding, the bead solution is co-flowed on either side of a wash buffer such that large beads migrate across streamlines towards the wash buffer located in the channel center due to the inertial forces, simultaneously achieving purification and rapid solution exchange with 100% bead collection efficiency [165,166].

**Figure 10:**
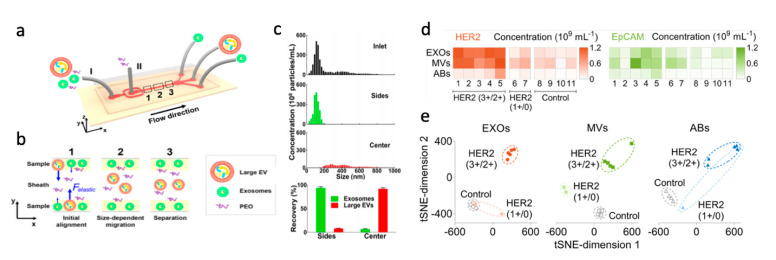
(**a**) Device using inertial microfluidics coupled with viscoelastic forces for EV fractionation. (**b**) At the inlet, both exosomes and microvesicles are in the sample flow, but as the EVs flow through the device, the larger EVs experience a larger viscoelastic force, pushing them into the sheath flow. (**c**) Representative graph of particle sizes at the inlet and the outlets (sides and center). The center outlet mainly has large EVs, while the side outlets contain purified exosomes. Panels (**a**–**c**) reprinted with permission from [168]. Copyright 2017 American Chemical Society. (**d**) Profiles of HER2 (left plot) and EpCAM (right plot) expression on different subpopulations of EVs, showing heterogeneity of EV expression. (**e**) Profiles of HER2 expression from exosomes (left plot), microvesicles (middle plot), and apoptotic bodies (right plot). Performing t-distributed stochastic neighbor embedding (t-SNE) reveals that HER2 expression on MVs from cancer patients share the least amount of overlap with EVs from healthy donors, compared to HER2 expression on exosomes and apoptotic bodies. Panels (**d**,**e**) reprinted with permission from [169]. Copyright 2019, American Chemical Society.

Other groups have utilized viscoelastic fluids to further enhance the inertial focusing on nanometer-sized extracellular vesicles to achieve label-free focusing and separation. Zhou et al. used polyethylene oxide (PEO) solution, a non-Newtonian fluid, in a series of alternating spiral microchannels to separate exosomes of interest from larger EVs [167]. Working in tandem with the viscoelastic forces of the PEO solution, the alternating spiral geometry provided reversing Dean forces on the sample. The large EVs (>300 nm) were focused into the sheath flow at the center of the channel, while smaller exosomes (~100 nm) remained in the flow along the side walls. This device separated 81% of exosomes with 95% purity from a sample of breast cancer cell culture medium, while a commercial kit achieved only 64% purity in the exosome isolate. Liu et al. combined a viscoelastic fluid with a straight channel geometry to focus and remove the large EVs in the sheath flow in the center of the channel, shown in Figure 10a,b [168]. Exosomes were recovered from pure fetal bovine serum (FBS) at a recovery rate of 80% with 94% purity (Figure 10c). Exosome isolation using this IM device requires an on-chip processing throughput of 200 μL/hr; this is highly efficient both in processing time and purity than other methods, including the gold standard ultracentrifugation (20 μL/hr, 5–25% recovery rate) and a microfluidic deterministic lateral displacement platform (12 nL/hr). 

Liu et al. also used a straight channel device that co-flows a Newtonian sheath buffer on either side of an EV sample suspended a highly concentrated λ-DNA solution with viscoelastic properties to separate exosomes, microvesicles, and apoptotic bodies [169]. The subpopulation recovery rates were 91%, 92%, and 89% for exosomes, microvesicles, and apoptotic bodies, respectively, with high purities of 96%, 94%, and 93%, respectively. EVs from Stage II breast cancer patients were labeled with aptamers targeting EpCAM and human epidermal growth factor receptor 2 (HER2), allowing for molecular analysis in addition to the size-based inertial separation of subpopulations. Quantification of these markers indicates that the HER2 and EpCAM concentrations were higher in all EV subpopulations for HER2 (3+/2+) patients than HER2 (1+/0) patients or controls (Figure 10d). A *t*-distributed stochastic neighbor embedding (*t*-SNE) analysis revealed that quantifying expression levels of cancer biomarkers on microvesicle subpopulations could be the most efficient for discriminating Stage II breast cancer patient cells from healthy donors (Figure 10e) [169].

Tay et al. utilized a spiral IM device and Dean vortices-induced migration to separate and purify synthetic and biological microparticles with applications in particle-based drug delivery systems and patient profiling [170]. Drug coated poly (lactic-co-glycolic acid) polymer particles ranging from 0.1–10 μm were separated into three groups with means diameters of 6.8 μm, 1.7 μm, and 0.89 μm, demonstrating single micron resolution. Utilizing the same device, the group was able to purify circulating EVs from patient blood. They found that Dean vortices-induced migration separates EVs from blood in a more timely and gentle manner than ultra-centrifugation, facilitating more reliable analysis. Using flow cytometry, they showed correlations between levels of immune cell-derived microparticles and common cardiovascular risk factors (body mass index, triglyceride levels) in T2DM patients [171,172].

Summary and Outlook

Subpopulations of EVs are receiving increasing attention for their potential roles as disease biomarkers and carriers of therapeutic materials. IM, combined with affinity methods or viscoelastic fluids, has shown the potential to assist in extracellular vesicle profiling for understanding diseases by segregating EV subpopulations with better purity and efficiency than that of conventional methods.

## 3. Exogenous Targets

Many diseases are caused by the invasion and multiplication of exogenous pathogenic microorganisms, including bacteria, fungus, viruses, and parasites, in the human body through the bloodstream, inhalation, or food and water consumption. Such pathogens are typically smaller (<2 µm) than mammalian cells, providing an opportunity for size-based inertial microfluidic (IM) enrichment and purification from contaminated samples. The enriched pathogens can be used in the downstream analysis for disease diagnosis or therapy. While there exist various innovative systems optimized using idealized samples, such as microbead suspensions, the scope of this review focuses on devices that have been validated with samples that more precisely replicate the heterogeneity of real-world samples, such as blood spiked with the target microorganism at clinically relevant concentrations. This is an important step in translational research to bring IM into the evolving field of microorganism detection for the patient and public health.

### 3.1. Bacteria and Fungus

Introduction

Effective treatment of bacterial or fungal bloodstream infections (BSIs) requires rapid identification of the disease-conferring organism and determination of appropriate therapeutic drugs to prevent the progression of the infection, which can lead to sepsis. Sepsis is systemic organ dysfunction and results in death for 30–50% of infected patients, even in the most advanced modern healthcare facilities [173]. Inertial microfluidics (IM) can play a role in both the diagnosis and treatment of bloodstream infections by isolating low-abundance pathogenic microorganisms for downstream analysis or by removing sepsis-causing pathogenic microorganisms and associated inflammatory biomolecules from patient blood, thereby mitigating progression to sepsis. The IM devices used with bacteria and fungus are outlined in Table 3.

Enrichment and Analysis of Bacteria

Rapid pathogen identification enables prompt selection of the correct drugs for treatment (e.g., antibacterial, antifungal, or antiviral) to avoid development of antibiotic resistance [174]. After the onset of sepsis, the likelihood of patient mortality increases by 7.6% with each hour that the patient is left untreated, making timely detection vital [119]. The current culture-based diagnostic methods involve multiple time-consuming growth stages and drug susceptibility assays that can take several days [174]. Molecular-based assays, including polymerase chain reaction (PCR) and other nucleic acid-based methods, are faster alternatives but require pre-processing of samples. Pathogen concentration in whole blood can be as low as 10–100 cfu/mL for sepsis, which may not be detectable by the assay without enrichment [175]. Additionally, whole blood contains many PCR-inhibiting blood cells [176]. IM presents the opportunity to quickly enrich bacteria and fungus from blood samples for downstream analysis based on the inherently smaller size of these pathogens compared to mammalian cells. Important measures to determine overall device performance are final purity, efficiency of pathogen recovery, enrichment factor, and throughput/processing time.

Several IM devices relied on diluted blood samples to maximize the final purity by reducing cell-to-cell interactions. Wu et al. isolated *Escherichia coli* from 20× diluted blood using a sheath flow in a straight channel [177]. This device requires more than 18 hours to process 1 mL of blood due to the large sample dilution and a low flow rate. While the process was only validated for high bacteria concentrations of 1.6 x 10^7^ cfu/mL, Wu et al. report 62% bacteria recovery, resulting in 300-fold bacteria enrichment with 99.71% purity. Hou et al. processed blood with clinically relevant pathogen loads (10^2^–10^4^ bacteria/mL) [178]. In just 20 minutes, bacteria were isolated from 1 mL of 3× diluted blood using a spiral microchannel. Bacteria recovery (>65%) was sufficient to identify the target bacteria species using ribosomal RNA (rRNA) sequences. The entire isolation and rRNA pathogen identification workflow took only 8 hours and produced results comparable to a conventional >48-h culture. Antibiotic resistance of the bacteria could also be assessed in less than 8 hours by analyzing mRNA transcripts, which requires a highly enriched sample due to the lower abundance of mRNA. Hou et al. paired a rapid IM enrichment method with nucleic acid-based detection and drug susceptibility analysis, emphasizing the ability of IM to streamline workflow for bacteria analysis.

**Figure 11:**
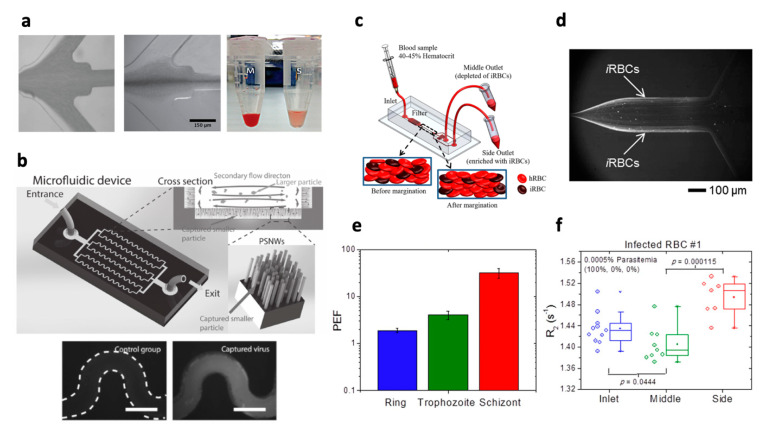
(**a**) The device performance for separation of RBCs without the use of Newtonian fluid (left) is compared to that with non-Newtonian fluid (center) and shows most of the RBCs exit through the center outlet only with the non-Newtonian fluid. On the right, a comparison of final samples collected in the middle outlet (M) and the side outlets (S). Adapted with permission from [176]. Copyright 2017, Springer Nature; permission conveyed through CC BY 4.0. (**b**) Symmetrically curved channels are used to create mixing to direct viruses toward the porous silicon nanowire (pSiNW) forest for capture. The control and captured channels show the comparison of no viruses and fluorescently labeled captured viruses (scale bar = 200 µm). Adapted with permission from [185]. Copyright 2016, John Wiley and Sons. (**c**) Schematic of the microfluidic device showing the focusing of infected RBCs (iRBCS) along the channel walls, while the healthy RBCs occupy most of the center of the channel. (**d**) Image showing fluorescently labeled iRBCs along the channels’ sidewalls. (**e**) Parasite enrichment factors (PEF) for various *Plasmodium* developmental stages. (**f**) Comparison of MMR measurements for the R_2_ relaxation rates of the inlet, middle outlet (healthy RBCs), and side outlets (iRBCs) show that the side outlets have significantly higher reading, indicating malaria infection. (**c**–**f**) Reprinted with permission from [188]. Copyright 2015, Springer Nature; permission conveyed through CC BY 4.0.

Eliminating pre-processing time for blood dilution, Faridi et al. utilized the synergistic effects of inertial forces and viscoelastic forces to isolate 76% of bacteria from undiluted whole blood with 92% purity [176]. *E. coli*-spiked blood was co-flowed on either side of a non-Newtonian fluid, causing the larger blood cells to migrate into the center non-Newtonian region, while the small bacteria cells remained aligned along the walls (Figure 11a). This proof-of-concept device requires about 17 hours for 1 mL of blood due to inherently low flow rate imposed by non-Newtonian fluid but processing time can be reduced by device parallelization.

Enrichment and Analysis of Fungus

The fungus can also cause BSIs that require rapid testing to inform doctors on choosing appropriate treatment plans. Identification of pathogenic fungus is just as important as bacterial detection but is often overlooked in early treatment. Fungal infections are typically treated with ineffective antibacterial drugs as a first defense, while lengthy blood cultures are processed to identify the specific pathogenic organism responsible for the infection [181]. This can lead to a significant delay in the administration of the proper drugs, worsening the outcomes. Fuchs et al. tested a spiral microchannel to fractionate small *Candida* cells, the most common pathogenic fungus genus, from diluted blood [181]. Fungal cells with a starting concentration of 1600 cells/mL were recovered at 44.6% efficiency with 5× volume reduction, an enrichment sufficient for off-chip PCR detection. The high flow rate resulted in a 125 minute processing time for pathogen isolation. Along with this sample preparation, PCR pathogen identification could be completed within 6 hours, allowing for timely drug selection and intervention.

Blood Cleansing

Bacterial or fungal pathogens in the bloodstream can cause sepsis by activating a cascade of inflammatory immune responses and causing organ dysfunction. While it is important to isolate pathogens to determine therapeutic options, extracorporeal blood purification can remove pathogens and infection-associated endotoxins as well as inflammatory molecules, including cytokines from the blood, to reduce inflammatory immune response [182]. Extracorporeal blood purification has shown improved clinical outcomes for sepsis patients by reducing mortality and the length of intensive care unit visits [180,182]. IM’s inherently high throughput and label-free purification make it an excellent tool for extracorporeal blood purification, which requires rapid purification of large volumes of blood.

Using a straight channel with two bifurcation stages, Hou et al. removed 80% and 90% of *E. coli* and *Saccharomyces cerevisiae* cells, respectively, and more than 80% of inflammatory platelets and leukocytes from undiluted blood [180]. Although the device operates at ~1 mL/hr per channel, the simple design allows for a 6-fold increase in throughput with parallelization. Mach and Di Carlo processed blood diluted to 0.5% (*v*/*v*), allowing for a higher flow rate of 8 mL/min in a device consisting of 40 parallel channels [179]. This straight channel device removed 80% of bacteria while retaining 90% of the initial RBCs. While this device yields extremely high throughput, it necessitates pairing with a hemoconcentrator before returning blood to the patient. The concentration of blood can be achieved using IM, eliminating the need for additional off-chip processing that could introduce contamination. Martel et al. demonstrated a tunable asymmetric curved channel as a blood concentration platform that is capable of volume reduction of 400× with 95% sample recovery efficiency and throughput of 4 mL/min [183]. Importantly, the flow rate matched that of conventional blood cleansing devices for integration. Integration of blood cleansing and concentrating devices could provide rapid blood purification with no sterility breaks needed to help sepsis patients to mitigate their infection and improve overall health outcomes.

Summary and Outlook

The separation and enrichment of bacteria and fungus from the blood through IM have prospective clinical applications in both the diagnosis and treatment of BSIs. Administration of correct antibacterial or antifungal drugs is becoming more important due to the increasing development of drug-resistant pathogens. IM sample preparation provides enrichment to increase the detection limits of molecular-based assays and enables faster diagnosis than conventional time-intensive culture-based methods. On-chip pathogen detection, including molecular or phenotypic assays, has been demonstrated, suggesting that future devices can incorporate IM enrichment and detection of pathogens in a single system, further reducing processing time and off-chip sample handling [175,184]. In addition to pathogen identification, IM enables extracorporeal blood purification to remove pathogens and inflammatory molecules in the blood for the treatment of sepsis. IM integrated with a hemoconcentrator allows for streamlining of blood cleansing and reconcentration in a sterile process as an alternative to hemodialysis or extracorporeal blood purification.

### 3.2. Viruses

Introduction

Diagnosis and treatment of viral infections require isolation of sub-micrometer viral particles and proteins from bodily fluids containing a large background of endogenous human cells. Direct manipulation of viral particles (20–400 nm) is challenging in inertial microfluidic (IM) because it is difficult to achieve sufficient shear gradient forces to influence nanoscale viruses [185,186]. Consequently, IM technologies focus on the indirect isolation of viruses [185,186] and clinically relevant antibodies [187] from blood samples by inertially focusing large blood cells for removal.

Virus Detection

Viral DNA and RNA detection methods for diagnosis of infection, typically quantitative polymerase chain reaction (qPCR) or genomic sequencing, are hampered by high background signals from human nucleic acids. Thus, isolation and enrichment of viruses is an inevitable preprocessing step for accurate analysis of viral DNA and RNA. By taking advantage of Dean Flow Fractionation (DFF), Choi et al. separated cytomegalovirus (CMV) particles from a suspension of human blood cells to reduce the amount of human DNA and improve the performance of downstream metagenomic sequencing [186]. This enrichment technique achieved a 16.5-and 6.3-fold increase in viral reads through metagenomic sequencing for blood and plasma samples, respectively, at clinically relevant viral loads (10^4^ copies/mL), as compared to unenriched samples. Label-free virus capture was also achieved by a hybrid device reported by Xia et al., which implemented a porous silicon nanowire (pSiNW) forest tuned to physically capture H5N2 influenza viruses [185]. Microvortices created by Dean flow were used to push submicrometer viruses toward the walls where they could be captured by the pSiNW forest at 50% capture efficiency at a flow rate of 8 μL/min (Figure 11b). The final collection of the virus is completed by slow degradation of the pSiNW forest. In 24 h, 60% of the captured viruses were released, yielding 29% total virus recovery, which was sufficient for off-chip virus cultivation and RT-qPCR detection.

Viral Biomarker Identification

Identification of viral antibodies and antigen-presenting cells (APCs) is useful for the discovery of diagnostic biomarkers and therapeutic antibodies. Conventional affinity-based isolation methods can be greatly accelerated with multiplexed IM separation of target-binding beads. Sarkar et al. demonstrated expedited isolation of rare HIV-antibodies and HIV-APCs from HIV+ patient blood using a spiral microchannel for DFF of various sized microbeads coated in molecules having an affinity for distinct targets (e.g., therapeutic antibodies or other proteins) with minimal cross-contamination [187]. In addition to HIV+ targets, this device has broader applications in other viral infectious diseases, such as Ebola, that require rapid isolation of proteins for diagnostics, antibody-based therapeutics, or vaccine development.

Summary and Outlook

IM devices exploit indirect or hybridized separation strategies to purify viral particles, which allows for simultaneous manipulation of microscale human cells and nanoscale viruses. Additionally, traditional techniques to analyze viral infection biomarkers can be accelerated through the incorporation of IM.

### 3.3. Parasites

Introduction

Microfluidics can be used to enrich bloodborne parasites that cause diseases by exploiting the intrinsic biophysical characteristics of the parasites or the parasite-infected RBCs, including polarizability, size, and deformability [174]. Various inertial microfluidic (IM) devices have been implemented for malaria, a parasitic disease that affects millions of people annually around the world, primarily in Sub-Saharan Africa. Malaria infection typically starts with a mosquito bite, which introduces a single-cell parasite of the genus *Plasmodium* to the bloodstream leading to infection of individual RBCs. After invading the RBC, the parasites mature over 48 hours in developmental stages: ring (early stage) followed by trophozoite and schizont (late stage), which also corresponds to changes in the RBC biophysical properties. The host RBC loses its distinct deformable biconcave disk shape and becomes stiffer and more spherical as the parasite progresses through the developmental stages [189,190]. The gold-standard diagnosis method, Giemsa-stained blood smear, and commonly used qPCR detection methods are time-consuming and require special skills and expensive equipment. These methods often result in a false negative due to low sensitivity, especially during the ring stage when blood parasitemia is low. For those infected with the most deadly species, *Plasmodium falciparum (P. falciparum)*, a false negative can be fatal because patients often die within 48 hours after showing their first symptoms [191].

Enrichment and Detection of Parasites

IM can improve detection accuracy by enriching parasites from infected whole blood using devices that can be deployed in resource-limited settings for rapid testing. IM relies on the inherent morphological differences among target and healthy cells to fractionate parasites and infected cells from blood samples. Various IM methods successfully separated the small *Plasmodium* parasite cells (~2 µm) from WBCs (6–15 µm) based on size or separate infected red blood cells (iRBCs) from healthy red blood cells (RBCs) based on shape and stiffness. Table 4 summarizes the design parameters used for IM devices, as well as their enrichment and separation capabilities.

Parasite enrichment from lysed blood has been demonstrated by performing size-based inertial separation of WBCs and *Plasmodium* parasites [191,192]. Warkiani et al. implemented a contraction-expansion array (CEA) to inertially focus WBCs for depletion at a flow rate of 400 μL/min [191]. They reported the removal of 99.99% of WBCs and recovery of 71% of parasites from lysed blood, containing clinically relevant concentrations of infected RBC (iRBC) at the ring stage (2–10 × 10^3^ iRBCs/mL). This parasite enrichment ultimately translated into a two-fold increase in the qPCR signal as compared to that from an unprocessed blood sample. Nam et al. suspended the iRBC-lysed blood sample in low viscosity non-Newtonian polymer solution and processed it in a straight, high aspect ratio channel to achieve sharper focusing of both WBCs and parasites [192]. This setup recovered 94% of parasites with 99% purity from samples with low initial parasitemia levels (500 parasites/mL) while maintaining high flow rates (400 μL/min). The parasite enrichment was sufficient for *P. falciparum* detection through PCR, which was not possible with the unenriched sample.

Alternatively, parasite enrichment can be performed from infected whole blood without lysing RBCs prior to processing. Healthy, deformable RBCs migrate to the center of microscale channels due to inertial lift forces and the Fahraeus-Lindqvist effect while spherical and larger cells, including iRBCs and WBCs, focus closer to the channel wall [190]. Based on these biophysical differences, Gieslinger et al. separated iRBCs from RBCs in a straight channel with the blood diluted to 2% hematocrit to reduce cell-to-cell interactions [189]. This method, although low throughput (40 μL/hr), resulting in an enrichment factor of 4.3 for ring stage iRBCs. Hou et al. enriched iRBCs from healthy RBCs in a straight channel, recovering ~75% of ring-stage iRBCs and >90% of schizont/trophozoite stage iRBCs from whole blood (a hematocrit of 40%) [190]. Although WBCs were also enriched through this process, Hou et al. suggested that larger WBCs could easily be removed on-chip through size-based micropillars installed at the device inlet. Kong et al. implemented a straight channel device to separate spiked iRBCs from buffy coat removed blood (a hematocrit of 45% and 0.0005% iRBC, Figure 11c,d) [188]. Parasite enrichment factors of 1.9, 4.1, and 32.1 were demonstrated for the ring, trophozoite, and schizont stages, respectively, based on the increasing stiffness associated with later developmental stages (Figure 11e).

Enrichment of *Plasmodium* parasites from blood samples is an important preparation step to increase the accuracy of diagnostic assays. PCR is a common detection method, but it may not be cost-effective to be used in resource-limited. Kong et al. developed a protocol for rapid infection detection using miniaturized magnetic resonance relaxometry (MRR) to be used in resource-limited settings [188]. iRBCs have relatively higher magnetic susceptibility than healthy RBCs because of hemozoin crystallites produced by *Plasmodium* parasites. This coupling of iRBC enrichment and multiple MRR measurements was able to detect ring parasites parasitemia as low as 0.0005% (Figure 11f). Although the cost of miniaturized MRR hardware is still several thousand dollars, individual assays cost less than 50 cents. Efficient enrichment of parasites through IM can allow for the use of novel, low-cost rapid diagnostic tests in the regions where malaria is more prevalent.

Biomarker Identification

IM can also provide a means to discover unique surface proteins of parasitic iRBCs for effective malarial diagnostics and therapeutics developments. Conventional methods for protein purification, such as immunoprecipitation, are time-consuming and insufficient for targeting iRBCs with few known malaria-specific surface proteins [193]. Birch et al. combined the benefits of IM with the SELEX (Systematic Evolution of Ligands by EXponential enrichment) method to identify aptamers that bind to epitopes expressed on iRBCs surfaces with high specificity. A sample with *P. falciparum* iRBCs was incubated with 10^14^ unique aptamers and injected into a spiral microchannel, separating aptamer-bound iRBCs from unbound aptamers in 10 minutes. This inertial separation was more efficient than conventional SELEX methods, removing 10^6^ unbound aptamers in a single pass through the device. The iRBC-bound aptamers were sequenced to characterize the surface proteins that were targeted with high affinity [193]. This method identified a subset of aptamers that may be used to develop malaria treatments or vaccines.

Summary and Outlook

Malaria infection requires immediate treatment accompanied by tests that can return results within <48 h. IM is capable of rapid sample enrichment to enable testing within the limited time frame after infection. In addition to malaria diagnostics, IM can help characterize the proteome of RBCs infected with *Plasmodium* parasites for the development of malaria treatments.

### 3.4. Pathogens in Environmental Samples and Foodstuff

Introduction

Pathogenic microorganisms commonly infect people through widely accessible transmission routes, including air, food, and water. Many infectious diseases that have a particularly strong impact on public health, including influenza A (H1N1), Middle East respiratory syndrome (MERS), severe acute respiratory syndrome (SARS), and more recently, SARS-CoV-2, are transmitted through aerosolized viruses [194,195]. Foods are also common carriers of pathogens and are particularly difficult to analyze due to highly variable structures across food products [196]. Similarly, water supplies are rigorously monitored public resources that still periodically cause waterborne illnesses even in countries with robust public health systems [197]. Rapid and routine sampling of air, food, and water can detect a pathogen before the broader public comes into contact with the contaminated resource, reducing the number of people affected by an outbreak. This is challenging, as pathogens can exist at very low concentrations in large volumes with high background signals from dust or food particles contamination; according to the World Health Organization, the maximum concentration of biological aerosols in indoor environments is 100–1000 cfu/m^3^ [198,199]. Common pathogen detection methods are time-consuming and require high concentrations of pathogenic microorganisms, rendering fast and early detection challenging. Inertial microfluidic (IM) devices improve the efficiency of sampling methods by providing high-throughput separation and concentration of low-abundance target pathogens.

Aerosol Sampling

The detection of bioaerosols in indoor environments is an important method for the prevention and control of pathogenic aerosolized viruses, bacteria, or fungal spores as they cause allergies, asthma, and other adverse reactions in humans [200]. Conventional bioaerosol sampling methods depend on the impaction of bioaerosol onto a rigid substrate, which suffers from high loss as particles rebound off the surface, non-specific collection, and high stress to the pathogen. Direct collection of pathogens into a liquid solution is preferred to preserve the pathogen integrity [201].

Hong et al. designed an inertial separator that can gently separate particles of three distinct size ranges. The device was designed with two sequential 90 curved air channels to separate targets into three outlets with Dean and centrifugal forces [194]. Large particles (>3 μm), such as dust, were removed in the first stage, and *Staphylococcus epidermidis* bacteria (1–2 μm) were separated from Adenovirus particles (<0.5 μm) in the second stage. Outlet samples were collected on gelatin filters and analyzed with qPCR to determine collection efficiency. 70% of the larger background particles were removed in the first stage. 67.1% of virus particles and 71.7% of the bacteria particles were separated into the appropriate outlets. High purity collection into three distinct outlets demonstrates the ability to differentiate between pathogen types as well as dust particles based on size.

**Figure 12:**
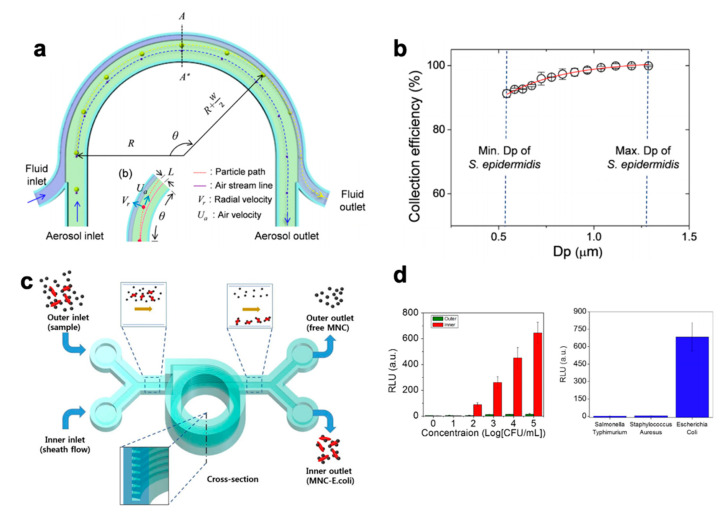
(**a**) The device schematic shows the curved channel and transfer of the aerosolized particles from gas to a fluid. (**b**) Device collection efficiency over the size range of S. epidermidis. (**a**,**b**) Reprinted with permission from [201]. Copyright 2017, American Chemical Society. (**c**) Microfluidic device design consists of stereolithography 3D printed, stacked spiral microchannel to separate free magnetic nanoparticles from the E. coli-bound particles. (**d**) ATP luminescence measurements show the limit of detection (100 cfu/mL) and organism binding specificity of this enrichment and detection protocol. (**c**,**d**) Reprinted by permission from Springer Nature: Scientific Reports [202]. Copyright 2015.

Choi et al. demonstrated rapid aerosol collection using a single 180° curved channel, shown in Figure 12a, that gently transferred target aerosols from the airflow to liquid stream for real-time detection [201]. The aerosol and collection liquid were injected through two different inlets, forming a stratified flow with the liquid phase on the outer wall of the curve and the gaseous phase on the inner wall. Bioaerosols were transferred from the inner gas phase to the outer liquid phase as the sample flowed through the curved microchannel. The collection efficiency of the device was >90% for aerosolized *S. epidermidis* bacteria (~0.84 μm), shown in Figure 12b, and different size ranges for collection could be targeted by adjusting the airflow rate. After a 10-minute collection period, a colony counting assay showed that the concentration of the collected bacteria was 6 times larger than that from the two conventional methods, a gelatin filter and a liquid-collection aerosol sampler. The collected bacteria also exhibited higher culturability than the conventional methods [201]. The collection of bacteria directly into a liquid solution allows for the device to be integrated with on-chip real-time detection systems.

Food Sampling

Early discovery of pathogens in food sources is important to prevent the spread of foodborne illness. Current testing methods, including PCR and immunofluorescence, are not widely adopted in the field because of low pathogen concentration and high background signals in food matrices. IM enables enrichment and purification of pathogens from food samples for fast and accurate detection.

Due to the high variability in food matrix types and consistencies, IM devices must be optimized for specific foods and target pathogens for adequate enrichment [196]. While food particles can inhibit antibody binding, Lee et al. successfully coupled immunomagnetic capture with IM to isolate *E. coli* from milk [202]. The mixture of milk and antibody-labeled magnetic nanoparticle clusters (MNCs) was injected at 5 mL/min into a spiral microchannel to separate free nanoparticles from the inertially focused *E. coli*-MNC complexes. To increase separation efficiency, the device consists of 10 stacked spiral microchannels with a constant radius of curvature and trapezoidal cross-sections, fabricated with stereolithography 3D printing (Figure 12c). Lee et al. used both a portable UV-Vis spectrometer and ATP luminescence measurements for the detection of bacteria concentrations as low as 100 cfu/mL (d) [202].

Clime et al. reported a straight channel device with three outlets to separate *Listeria monocytogenes* bacteria from a suspension of ground beef debris particles [203]. At a flow rate of 130 μL/min, 70% of the bacteria was recovered, and 40% of the food debris was removed. Ganz et al. isolated the parasite *Giardia duodenalis* spiked in commercially purchased lettuce products by inertially focusing cysts in a straight channel with a pumped 6-cycle recirculating setup [196]. For a 10 mL sample, the system achieved 7-fold enrichment in 28 minutes. Cytometry measurements showed 10-fold greater food debris removal achieved by the IM method than the conventional filtration-centrifugation method.

Water Sampling

Water sources can be contaminated with pathogenic microorganisms, especially by those that are difficult to detect or resistant to standard water treatments [197]. Detection of pathogens in large volumes of water and requires several intensive filtration, elution, and centrifugation steps to concentrate and identify various microorganisms. IM separation can replace these time-consuming processes to isolate of pathogens at low concentrations. Jimenez et al. used a spiral microchannel to isolate the waterborne pathogens, *Cryptosporidium parvum* and *Giardia lamblia*, from water samples [197,204]. The device captured 95% of *C. parvum* and 83% of *G. lamblia* at a flow rate of 1 mL/min. Parallelization of the device allowed for an 80-fold volume reduction in just 10 minutes [204].

Summary and Outlook

To fully employ IM for a routine sampling of air, food, and water, IM pathogen enrichment methods have been integrated with fast and simple off-chip detection techniques, including UV-vis spectroscopy and bioluminescence measurements done by portable devices [196]. IM pathogen enrichment can also be combined with and improve the limits of detection of microfluidic pathogen detection platforms that rely on optical, electrochemical, biophysical and other detection methods [184].

## 4. Hybrid Systems for New Capabilities

Simple design and ease of operation make IM widely integrable with other technologies to form “hybrid devices” capable of additional in-line or on-chip microfluidic manipulation. These devices offer improved enrichment efficiency or novel functionalities compared to standalone techniques. The integration of different functionalities can remove pre- and post-processing steps, making the addition of IM devices to current pipelines more attractive.

Existing hybrid devices use added complexities to improve sample target enrichment. Xia et al. demonstrated the capture and release of nanoscale viruses inertially mixed in a degradable nanowire forest fabricated on three walls of a microfluidic chip [185]. The combination of these technologies enabled label-free virus isolation and improved the resolution of virus separation. Choi et al. implemented a curved channel with a two-phase flow to capture and transfer airborne bacterium from aerosol samples into a liquid solution [201]. This technique addressed common limitations of liquid-based bacteria collection by improving recovery and providing compatibility with real-time detection methods. Ramachandraiah et al. and Zhu et al. used spiral microchannels to isolate nucleated cells and WBCs, respectively, from blood samples after lysing RBCs on-chip [100,101]. Critically, these devices preserved cell viability through gentler, faster processes relative to conventional, density-based blood purification techniques and yielded high separation efficiency by reducing the manual steps in the protocol.

Hybrid devices hold promise in expanding the utility of IM with novel functionalities. Vortex and spiral microfluidic chips have been paired with impedance profiling for label-free quantification of isolated CTCs and WBCs [98,205]. The coupling of these microfluidic devices enables rapid enumeration and size characterization while preserving cells for further analysis. Ouyang et al. developed a hybrid vortex device to trap and electroporate target cells using gold-plated electrodes [206]. The device leverages rapid solution exchange and cell trapping for multimolecular delivery with higher transfection efficiencies than conventional electroporation methods. Moon et al. improved single-cell RNA sequencing by using a hybrid device to inertially focus barcoded beads prior to droplet formation [207]. The deterministic nature of inertial focusing helped improve the fraction of single-bead-per-droplet encapsulation and minimize errors typically associated with Drop-Seq. Dhar et al. developed a hybrid device capable of isolating CTCs and encapsulating trapped cells in microdroplets to study single-cell protease activity [75]. The hybridization was necessary to minimize rare cell loss, decrease cell processing time, and lower natural background protease concentrations from bodily fluids.

Beyond these existing devices, there remains ample room for further development of hybrid devices with clinical benefits. Following the broader goal of ‘plug-and-play’ standardization of microfluidics, IM can be integrated with already established microfluidic devices. Microfluidic RBC lysis devices can be positioned upstream of other microfluidic tasks, such as CTC isolation or cell stretching [53,100,101,102,103], that require sample preprocessing. Similarly, future studies can build on the integration of target isolation and droplet generation for single-cell analysis [208]. The further development of hybrid devices will improve microfluidic device performance and enable new capabilities.

## 5. Conclusions

Inertial microfluidic (IM) technologies deliver on their promise of label-free, high-throughput sample processing. These devices are compatible with easily obtainable samples, such as blood, urine, and mucus, which allows for routine monitoring of patient status. IM has the clinical utility to reduce the processing workload during a analysis of raw samples.

These devices are capable of isolating rare cells based on size, facilitating further analyses that provide invaluable insight into the diseased state of patients. IM purification efficiency is higher than the current CTC enrichment gold standards and has enabled investigations of tailored therapeutic options for individual patients. IM devices have been used to separate specific subpopulations of WBCs, without the aid of biomarkers and labeling agents, thus preserving the integrity of the samples and increasing the reliability of downstream tests. Size and deformability-based assays have improved the sensitivity of diagnostics by assaying thousands of cells with single-cell resolution in a clinically relevant timeframe. By setting a minimum separation threshold, all the cells from a sample can be removed, producing analyte rich supernatants. IM can even separate nano-sized bioparticles from heterogeneous samples when non-Newtonian viscoelastic solutions are used. Pathogenic microorganisms, which also tend to be smaller than human cells, can be rapidly enriched for time-sensitive detection assays and biomarkers studies.

Future Directions

Presently, reported IM devices are poised to make a positive impact in clinical and public health applications. While findings from the reported works are promising, many studies use devices fabricated and operated with different protocols, contributing to user-to-user variation. Future work should focus on improving device capabilities and usability and taking steps towards practical, widespread usage and standardization of IM technologies in clinical settings. Advances in additive manufacturing have enabled creative 3D geometries that enhance inertial effects to improve purification efficiency and are not limited by traditionally planar microfabrication techniques [202,209]. The integration of IM with other microfluidic technologies to produce hybrid devices will lead to new, improved assay performance. As the design of these devices improves, it is critical to standardize experimental hardware and software to translate these technologies out of research laboratories. Several companies, including Vortex Biosciences, Cytovale, and Clearbridge BioMedics, are already commercializing and standardizing IM devices. These companies are working towards integrating IM devices into sample preparation workflows for clinical research, with the eventual goal of augmenting or replacing current procedures. Integration, standardization, and commercialization will ultimately lead to more regular use of IM in clinical settings. In all, IM devices apply simple working principles towards critical biological targets and have demonstrated the ability to positively impact patient and public health as a translational technology.

## Figures and Tables

**Table 1:** Summary of literature on IM based WBC separation and assaying, discussed in this review paper. Key features highlighted are: channel geometry, sample type, WBC target, dilution factor, and throughput. References listed for further detail.

Channel Geometry	Sample	Target	Dilution Factor	Throughput (min) *	Reference
Spiral	Tracheal secretions	Neutrophils	1000×	250	[95]
Spiral	Blood	Granulocytes	1000×	250	[94]
Spiral	Blood	Leukocytes	10×	76.9	[96]
Spiral	Blood	Neutrophils and monocytes	1000×	6250	[97]
Spiral	Blood	Neutrophils	500×	3125	[98]
Spiral	Blood	Neutrophils	5×	50	[99]
Spiral	Blood	Leukocytes	14×	5.83	[100]
Spiral	Blood	All subpopulations	10×	10	[101]
Asymmetrically curved	Blood	Neutrophils	N/A **	N/A **	[102]
Asymmetrically curved	Pleural fluid	Granulocytes	N/A **	N/A **	[103]

* defined as min per 1 mL of undiluted sample volume, calculated as (dilution factor/flow rate). ** Samples were collected, and hemolysis performed, resulting in WBCs. Dilution factor and thus throughput could not be determined.

**Table 2:** Summary of literature on inertial microfluidic plasma extraction investigated in this review paper. Key performance metrics, plasma purity, dilution factor, throughput, extraction efficiency, and channel design are listed with references.

Channel Design	Plasma Purity	Dilution Factor *	Throughput **	Extraction Efficiency	Reference
Contraction-expansion array in a straight channel	92.60%	1 ^a^	33.3	69.50%	[148]
Microchannel with the slanted groove structure	99.90%	1 ^a,b^	2 ^b^	Not specified	[154]
Serpentine channel with a membrane filter	100%	20	7.1	Not specified	[159]
Spiral microchannel	~100%	20	28.6	38.50%	[151]
Spiral microchannels with micropillar filters	Not clear ^c^	20	2000	53.90%	[156]
Spiral microchannel with a trapezoidal cross-section	100%	45 or 90	60 or 120	Not specified	[152]
Spiral microchannel with microbar obstacles	99.96%	18	6	67.60%	[153]
Spiral channel with a low-aspect-ratio cross-section and microbar obstacles	99.99%	15	3-15	67.57%	[157]

* based on 45% hematocrit level; ** defined as min per undiluted sample volume, calculated as 1 mL/(flow rate/dilution factor); ^a^ Sheath flow is used; ^b^ based on 42% hematocrit level; ^c^ described as “below the threshold of the testing.”.

**Table 3:** Summary of parameters and performance of devices used for bacteria and fungus separation.

Channel geometry	Target	Dilution Factor	Throughput *	Pathogen load (/mL)	Separation Efficiency	Purity	Reference
straight	*E. coli*	undiluted	33 hr	10^6^	76%	92%	[176]
straight	*E. coli*	20×	18 hr	1.6x10^7^	62%	99.71%	[177]
straight	*E. coli*	90×	11.25 min	10^8^	80%	Not specified	[179]
straight	*E. coli* and *S. cerevisiae*	undiluted	10 min	10^6^–10^7^	80% (*E. coli*)*,* 90% (*S. cerevisiae)*	Not specified	[180]
spiral	*C. albicans/Candida*	10×	25 min	1600	44.60%	Not specified	[181]
spiral	*E. coli, K. pneumoniae*, *P.aeruginosa*, and *S. aureus*	3×	20 min	10^2^–10^4^	>65%	Not specified	[178]

* Throughput is defined as the time needed to process 1 mL of undiluted blood, calculated as 1 mL/(flow rate/dilution).

**Table 4:** Summary of parameters and performance of devices used for parasite enrichment.

Channel Shape	Sample	Hematocrit	Flow Rate (µL/min)	Pathogen Load	Parasite Enrichment Factor	Separation Efficiency	Reference
straight	*P. falciparum* iRBCs (all stages)	45%	10	0.0005–5% parasitemia	1.9 (ring) 4.1 (trophozoite) 32.1 (schizont)	Not specified	[188]
straight	*P. falciparum*	45%	400	500 /mL	6.4	94%	[192]
straight	*P. falciparum* iRBCs (all stages)	40%	5	0.01–1% parasitemia	2	75% (ring) >90% (trophozoite/schizont)	[190]
straight	*P. falciparum* iRBCs (ring stage)	2%	0.67	1.6–3.4% parasitemia	4.3	Not specified	[189]
CEA	*P. falciparum* (ring stage)	~10%	400	10^3^–10^4^ /mL	Not specified	70.9%	[191]
